# Crystal structures of two solvated 2-aryl-3-phenyl-2,3-di­hydro-4*H*-pyrido[3,2-*e*][1,3]thia­zin-4-ones

**DOI:** 10.1107/S2056989019013781

**Published:** 2019-10-22

**Authors:** Hemant P. Yennawar, Eric N. Thompson, Jennie Li, Lee J. Silverberg

**Affiliations:** a Department of Biochemistry and Molecular Biology, 108 Althouse Laboratory, Pennsylvania State University, University Park, PA 16802, USA; b Pennsylvania State University, Schuylkill Campus, 200 University Drive, Schuylkill Haven, PA 17972, USA

**Keywords:** crystal structure, pyrido­thia­zinone, envelope pucker, C—H⋯π inter­actions

## Abstract

The synthesis and crystal structures of 2-(4-fluoro­phen­yl)-3-phenyl-2,3-di­hydro-4*H*-pyrido[3,2-*e*][1,3]thia­zin-4-one toluene hemisolvate (**1**), C_19_H_13_FN_2_OS·0.5C_7_H_8_, and 2-(4-nitro­phen­yl)-3-phenyl-2,3-di­hydro-4*H*-pyrido[3,2-*e*][1,3]thia­zin-4-one iso­propanol 0.25-solvate 0.0625-hydrate (**2**), C_19_H_13_N_3_O_3_S·0.25C_3_H_7_O·0.0625H_2_O, are reported. Both are racemic mixtures (centrosymmetric crystal structures) of the individual com­pounds and incorporate solvent mol­ecules in their structures. Compound **2** has four thia­zine mol­ecules in the asymmetric unit. All the thia­zine rings in this study show an envelope pucker, with the C atom bearing the substituted phenyl ring displaced from the other atoms. The phenyl and aryl rings in each of the mol­ecules are roughly orthogonal to each other, with dihedral angles of about 75°. The extended structures of **1** and **2** are consolidated by C—H⋯O and C—H⋯N(π), as well as T-type (C—H⋯π) inter­actions. Parallel aromatic ring inter­actions (π–π stacking) are observed only in **2**.

## Abstract 2

The synthesis and crystal structures of two solvated 2-aryl-3-phenyl-2,3-di­hydro-4*H*-pyrido[3,2-*e*][1,3]thia­zin-4-ones are reported. Both are racemic mixtures (centrosymmetric crystal structures) of the individual com­pounds and incorporate solvent mol­ecules in their structures.

## Chemical context   

Compounds with a 2,3-di­hydro-4*H*-pyrido[3,2-*e*][1,3]thia­zin-4-one scaffold have shown anti­cancer (Arya *et al.*, 2014 ▸), anti­bacterial (Shreedhara *et al.*, 2017 ▸), and glycosidase inhibitory (Li *et al.*, 2012 ▸) bioactivity. These com­pounds feature a pyridine ring fused to a thia­zine ring. Previously, we reported the synthesis and structure of 2,3-diphenyl-2,3-di­hydro-4*H*-pyrido[3,2-*e*][1,3]thia­zin-4-one (Yennawar *et al.*, 2014 ▸). Herein, we report the syntheses and structures of two solvated analogs containing a substituent on the *C*-phenyl ring: 2-(4-fluoro­phen­yl)-3-phenyl-2,3-di­hydro-4*H*-pyrido[3,2-*e*][1,3]thia­zin-4-one as a toluene hemisolvate, **1**, and 2-(4-nitro­phen­yl)-3-phenyl-2,3-di­hydro-4*H*-pyrido[3,2-*e*][1,3]thia­zin-4-one as a mixed propanol–water solvate, **2**.
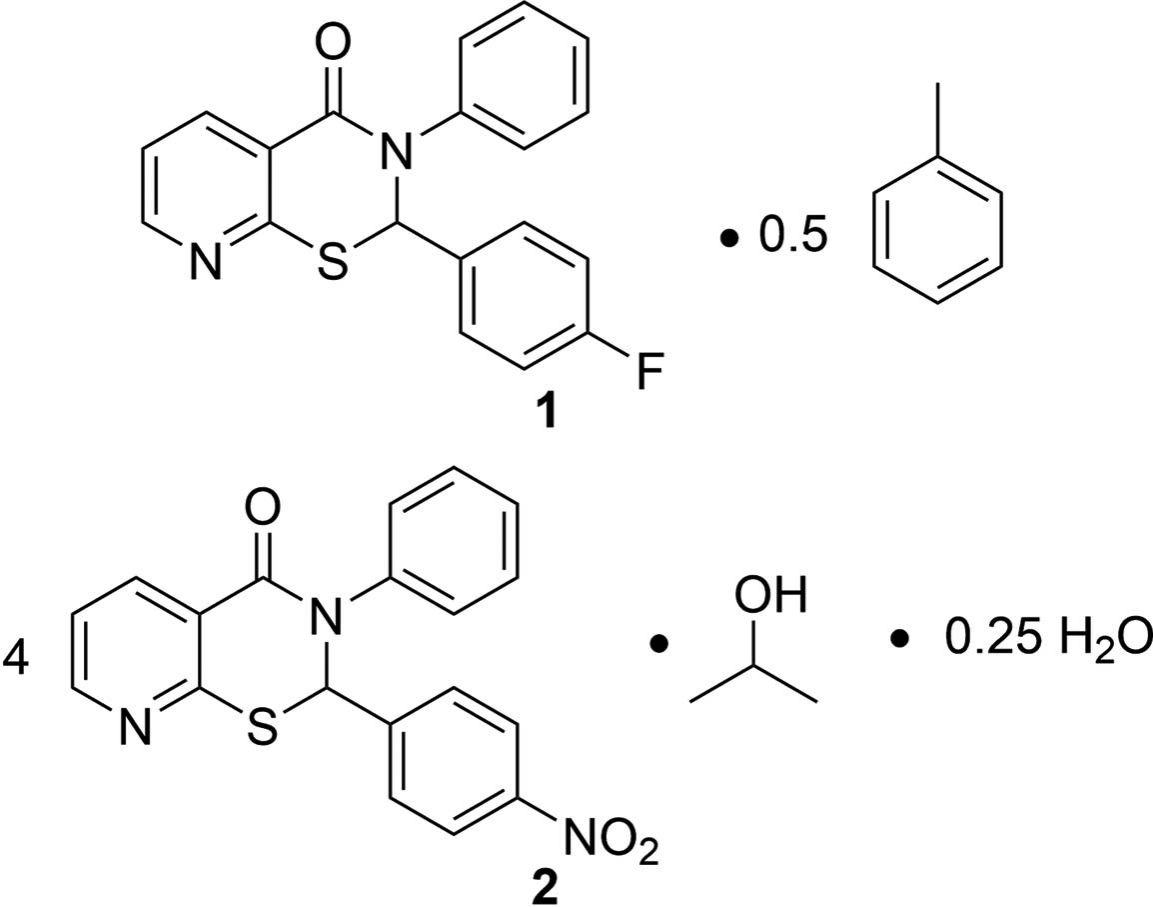


## Structural commentary   

**Figure 1:**
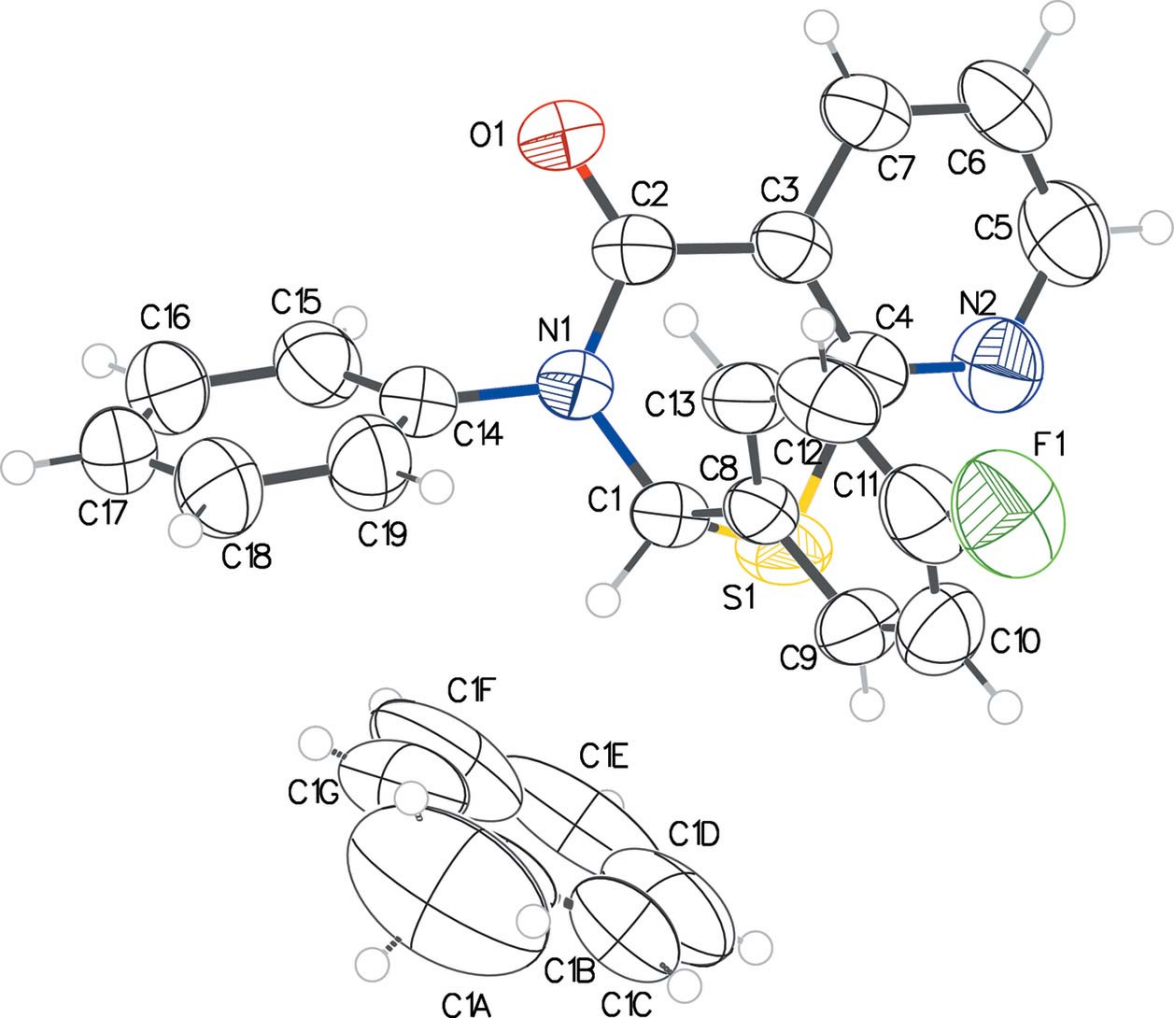
The asymmetric unit of **1** with the solvent toluene mol­ecule straddling the inversion center. The displacement ellipsoids are drawn at the 50% probability level.

The asymmetric unit of **1** (Fig. 1 ▸) com­prises the title mol­ecule along with the solvent mol­ecule (toluene) straddling an inversion center. The 1,3-thia­zine ring is in an envelope pucker conformation according to the puckering parameters [*Q* = 0.6016 (16) Å, θ = 115.35 (16)°, and φ = 220.50 (18)°] calculated by *PLATON* (Spek, 2009 ▸), with atom C1 displaced from the other atoms. The phenyl rings on the 2 and 3 positions of the thia­zine ring are close to orthogonal, forming a dihedral angle of 77.65 (10)°; their dihedral angles with respect to the N2 pyridine ring are 81.45 (11) and 79.22 (9)°, respectively. Atom C1 is a stereogenic center; in the arbitrarily chosen asymmetric unit, it has an *S* configuration, but crystal symmetry generates a racemic mixture.

**Figure 2:**
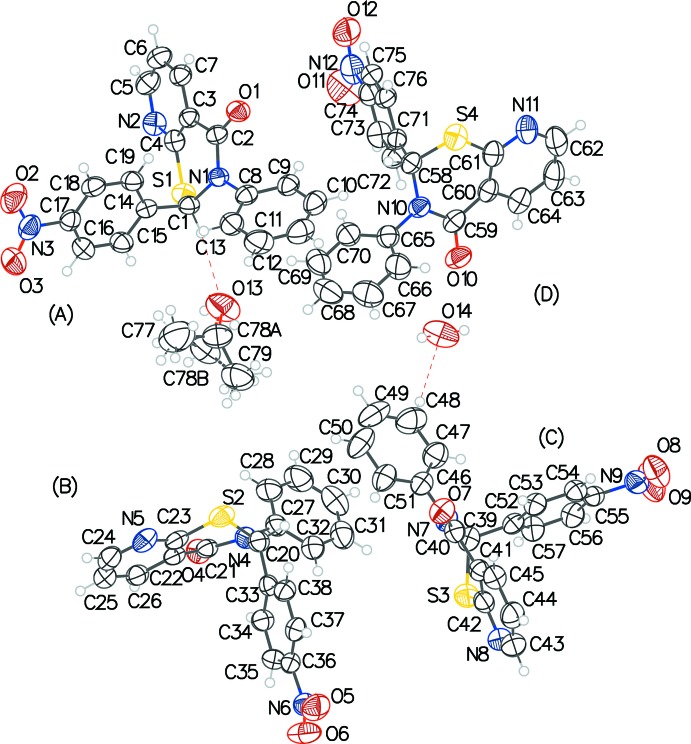
The asymmetric unit of **2** with solvent 2-propanol and water (0.25 occupancy) mol­ecules. The displacement ellipsoids are drawn at the 50% probability level.

**Figure 3:**
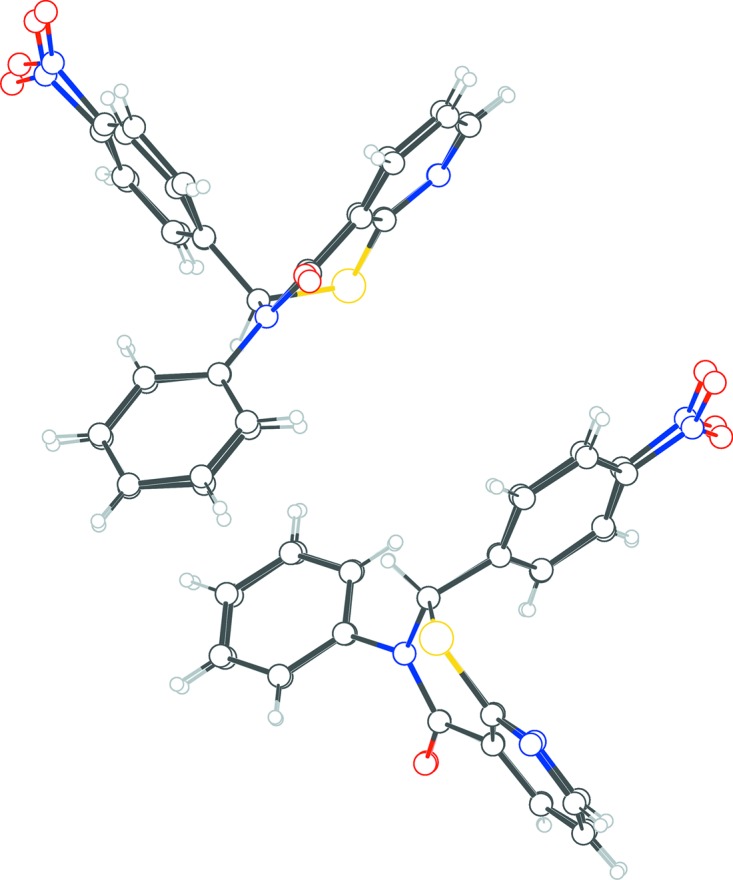
Overlay image for two pairs of enanti­omers in the asymmetric unit of **2**, showing the overall structural similarity.

**Figure 4:**
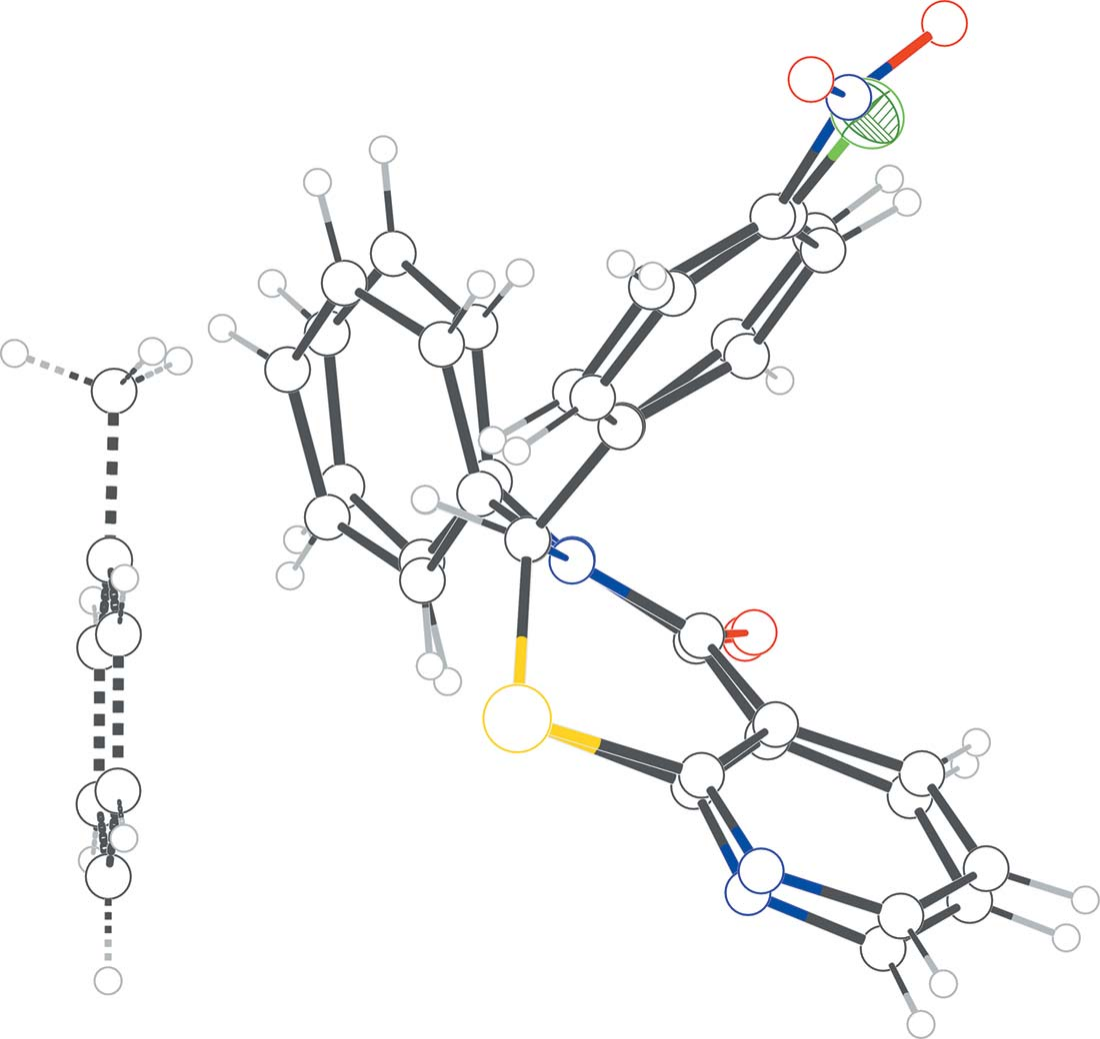
Overlay image showing the similarity of the structures of **1** and **2**.

In **2**, the configurations of the stereogenic centers in the four arbitrarily chosen independent thia­zine mol­ecules *A*, *B*, *C*, and *D* (Fig. 2 ▸) are *R* at C1 and C39, and *S* at C20 and C58. A solvent mol­ecule of 2-propanol and a water mol­ecule with partial (0.25) occupancy com­plete the asymmetric unit. The puckering of the thia­zine ring in each case is an envelope (*Q* ∼ 55 Å, θ ∼ 65°, and φ ∼ 41°, considering chirality tranformations), which is very similar to that in **1**. The four mol­ecules within this structure are all very similar in their three-dimensional dispositions, as can be seen in the overlay figure (Fig. 3 ▸). For the *X* (pyridine C3–C7/N2), *Y* (phenyl C8–C13), and *Z* (*para*-nitro­phenyl C14–C19) rings in mol­ecule *A*, the *X*/*Y*, *X*/*Z*, and *Y*/*Z* dihedral angles are 70.52 (16), 87.25 (14), and 89.09 (16)°, respectively. Equivalent data for mol­ecule *B* are 83.73 (16), 86.23 (14) and 77.44 (16)°, respectively; for mol­ecule *C* are 65.92 (17), 85.94 (14), and 85.84 (17)°, respectively; for mol­ecule *D* are 85.84 (18), 82.77 (14), and 77.72 (18)°, respectively. The superimposition of the structures of **1** and **2** (Fig. 4 ▸) also shows very little discrepancy.

## Supra­molecular features   

**Figure 5:**
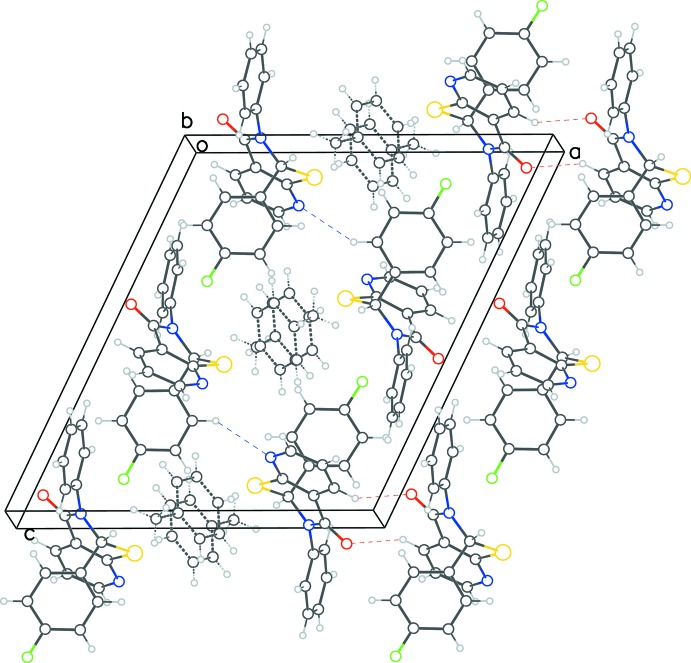
Packing diagram for **1**, showing the C—H⋯N(π) and C—H⋯O ‘reciprocal-pair’ of hydrogen bonds.

The asymmetric unit of **1** has the chiral C atom (C1) participating in a C—H⋯π-type inter­action with the toluene ring [C—H⋯π = 3.735 (3) Å, 142°]. The O atom on the fused thia­zine ring system accepts a C—H⋯O hydrogen bond from a symmetry-related pyridine ring in a parallel-reciprocal fashion (Table 1 ▸ and Fig. 5 ▸). Some other weak C—H⋯π inter­actions may help to consolidate the structure. The aryl and pyridyl rings of symmetry-related mol­ecules exhibit a T-type inter­action. No π–π parallel stacking is observed in this structure.

**Figure 6:**
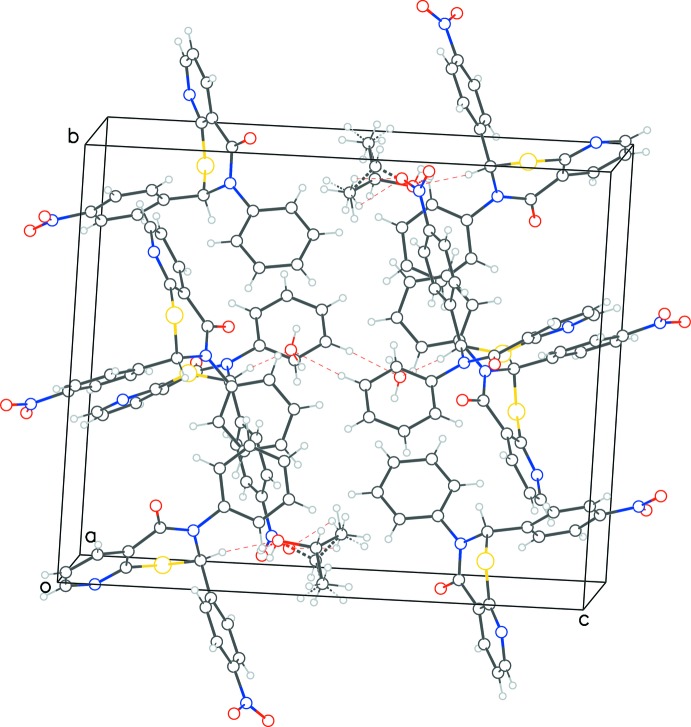
Packing diagram for **2**, showing the aromatic ring stacking interactions along with the C—H⋯N(π) and C—H⋯O hydrogen bonds.

Within the asymmetric unit of **2** the ‘3-phenyl rings’ of neighboring enanti­omeric mol­ecules (two pairs) exhibit a parallel stacking inter­action. One of the four phenyl rings participates in a C—H⋯O-type inter­action with the partially occupied water O atom (Table 2 ▸). The chiral C atom of mol­ecule *A* participates in a C—H⋯O hydrogen bond with the O atom of the solvent 2-propanol mol­ecule. The extended packing (Fig. 6 ▸) shows neighboring mol­ecules inter­acting *via* parallel stacking inter­actions between the phenyl rings, as well as between the aryl and pyridyl rings. The T-type ring inter­actions are also observed between the phenyl and aryl rings, as well as between the pyridyl rings of neighboring mol­ecules. Various C—H⋯O, O—H⋯O, and C—H⋯N type hydrogen bonds consolidate the structure.

## Database survey   

Along with the previously mentioned structure (Yennawar *et al.*, 2014 ▸), we have published a structure of the sulfoxide derivative (Yennawar *et al.* 2017 ▸). No other similar structures were found.

## Synthesis and crystallization   

For the preparation of 2-(4-fluoro­phen­yl)-3-phenyl-2,3-di­hydro-4*H*-pyrido[3,2-*e*][1,3]thia­zin-4-one (**1**), a two-necked 25 ml round-bottomed flask was oven-dried, cooled under N_2_, and a stir bar was added. The flask was charged with aniline (0.559 g, 6 mmol) and 4-fluoro­benzaldehyde (0.744 g, 6 mmol), and stirred for 5 min. Thio­nicotinic acid (0.931 g, 6 mmol) and 2-methyl­tetra­hydro­furan (2.3 ml) were added. Pyridine (1.95 ml, 24 mmol) was added and, finally, 2,4,6-tripropyl-1,3,5,2,4,6-trioxatri­phospho­rinane-2,4,6-trioxide (T3P) in 2-methyl­tetra­hydro­furan (50 wt%; 7.3 ml, 12 mmol) was added. The reaction was stirred at room temperature, followed by thin-layer chromatography (TLC), and then poured into a separatory funnel with di­chloro­methane (20 ml). The mixture was washed with water (10 ml). The aqueous fraction was then extracted twice with di­chloro­methane (10 ml each). The organics were combined and washed with saturated sodium bicarbonate (10 ml) and then saturated sodium chloride (10 ml). The organic fraction was dried over sodium sulfate and concentrated under vacuum to give a crude mixture, which was chromatographed on 30 g flash silica gel with mixtures of ethyl acetate and hexa­nes (30 to 70% ethyl acetate) to give a solid. Recrystallization from a solvent mixture of toluene and hexa­nes gave colorless crystals of **1** (yield 0.5705 g, 28%; m.p. 127.2–127.4 °C).

2-(4-nitro­phen­yl)-3-phenyl-2,3-di­hydro-4*H*-pyrido[3,2-*e*][1,3]thia­zin-4-one (**2**) was prepared by the same method with 1-(4-nitro­phen­yl)-*N*-phenyl­methanimine (1.35 g, 6 mmol) replacing aniline and 4-fluoro­benzaldehyde. The crude solid after the extractive workup was recrystallized from a 2-propanol solution to give colorless crystals of **2** (yield 1.3581 g, 62%; m.p. 119–121 °C).

## Refinement   

Crystal data, data collection and structure refinement details are summarized in Table 3 ▸. H atoms were positioned geometrically (C—H = 0.93–0.98 Å and O—H = 0.82 Å) and refined using a riding model, with *U*_iso_(H) = 1.2 or 1.5*U*_eq_(C,O).

## Supplementary Material

Crystal structure: contains datablock(s) global, 2, 1. DOI: 10.1107/S2056989019013781/hb7856sup1.cif

Supporting information file. DOI: 10.1107/S2056989019013781/hb78561sup4.mol

Structure factors: contains datablock(s) 1. DOI: 10.1107/S2056989019013781/hb78561sup6.hkl

Structure factors: contains datablock(s) 2. DOI: 10.1107/S2056989019013781/hb78562sup3.hkl

Supporting information file. DOI: 10.1107/S2056989019013781/hb78562sup5.mol

CCDC references: 1958350, 1958351, 1958350, 1958351

Additional supporting information:  crystallographic information; 3D view; checkCIF report

## Figures and Tables

**Table 1:** Hydrogen-bond geometry (Å, °) for **1** [Chem scheme1]

*D*—H⋯*A*	*D*—H	H⋯*A*	*D*⋯*A*	*D*—H⋯*A*
C7—H7⋯O1^i^	0.93	2.53	3.321 (2)	143
C9—H9⋯N2^ii^	0.93	2.58	3.398 (2)	147

Symmetry codes: (i) 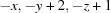; (ii) 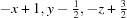.

**Table 2:** Hydrogen-bond geometry (Å, °) for **2** [Chem scheme1]

*D*—H⋯*A*	*D*—H	H⋯*A*	*D*⋯*A*	*D*—H⋯*A*
C1—H1⋯O13	0.98	2.49	3.461 (4)	172
C6—H6⋯N5^i^	0.93	2.74	3.431 (4)	132
C39—H39⋯O14^ii^	0.98	2.41	3.339 (10)	158
C44—H44⋯N11^iii^	0.93	2.77	3.479 (4)	134
C48—H48⋯O14	0.93	2.29	3.065 (12)	141
C50—H50⋯O2^iv^	0.93	2.62	3.412 (5)	144
C58—H58⋯O7^v^	0.98	2.49	3.246 (3)	134
C76—H76⋯O7^v^	0.93	2.78	3.498 (4)	135
C79—H79*A*⋯O9^ii^	0.96	2.57	3.353 (6)	138
C79—H79*E*⋯O8^vi^	0.96	2.61	3.455 (5)	147
C79—H79*F*⋯O2^iv^	0.96	2.75	3.493 (6)	135
O13—H13*B*⋯O4^vii^	0.82	1.95	2.766 (3)	176

Symmetry codes: (i) 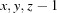; (ii) 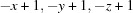; (iii) 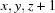; (iv) 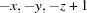; (v) 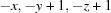; (vi) 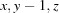; (vii) 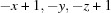.

**Table 3:** Experimental details

	**1**	**2**
Crystal data		
Chemical formula	C_19_H_13_FN_2_OS·0.5C_7_H_8_	4C_19_H_13_N_3_O_3_S·C_3_H_8_O·0.25H_2_O
*M*_r_	382.44	1518.13
Crystal system, space group	Monoclinic, *P*2_1_/*c*	Triclinic, *P* 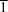
Temperature (K)	298	298
*a*, *b*, *c* (Å)	14.4481 (16), 9.0141 (10), 16.3427 (19)	12.5451 (13), 15.9804 (17), 19.434 (2)
α, β, γ (°)	90, 115.481 (2), 90	86.671 (2), 72.369 (2), 74.167 (2)
*V* (Å^3^)	1921.4 (4)	3570.8 (6)
*Z*	4	2
Radiation type	Mo *K*α	Mo *K*α
μ (mm^−1^)	0.19	0.21
Crystal size (mm)	0.27 × 0.26 × 0.26	0.19 × 0.18 × 0.03

Data collection		
Diffractometer	Bruker CCD area detector	Bruker CCD area detector
Absorption correction	Multi-scan (*SADABS*; Bruker, 2001 ▸)	Multi-scan (*SADABS*; Bruker, 2001 ▸)
*T*_min_, *T*_max_	0.877, 0.9	0.230, 0.9
No. of measured, independent and observed [*I* > 2σ(*I*)] reflections	14808, 4590, 3093	30898, 16372, 7969
*R*_int_	0.026	0.039
(sin θ/λ)_max_ (Å^−1^)	0.667	0.668

Refinement		
*R*[*F* ^2^ > 2σ(*F* ^2^)], *wR*(*F* ^2^), *S*	0.047, 0.151, 1.01	0.057, 0.158, 0.95
No. of reflections	4590	16372
No. of parameters	266	997
No. of restraints	57	30
H-atom treatment	H-atom parameters constrained	H-atom parameters constrained
Δρ_max_, Δρ_min_ (e Å^−3^)	0.27, −0.22	0.28, −0.27

Computer programs: *SMART* (Bruker, 2001 ▸), *SAINT* (Bruker, 2001 ▸), *SHELXS* (Sheldrick, 2008 ▸), *SHELXL2018* (Sheldrick, 2015 ▸) and *OLEX2* (Dolomanov *et al.*, 2009 ▸).

## supplementary crystallographic information

### 2-(4-Fluorophenyl)-3-phenyl-2,3-dihydro-4H-pyrido[3,2-e][1,3]thiazin-4-one toluene hemisolvate (1) Crystal data

**Table:**

C_19_H_13_FN_2_OS·0.5C_7_H_8_	*F*(000) = 796
*M**_r_* = 382.44	*D*_x_ = 1.322 Mg m^−^^3^
Monoclinic, *P*2_1_/*c*	Mo *K*α radiation, λ = 0.71073 Å
*a* = 14.4481 (16) Å	Cell parameters from 4037 reflections
*b* = 9.0141 (10) Å	θ = 2.5–28.1°
*c* = 16.3427 (19) Å	µ = 0.19 mm^−^^1^
β = 115.481 (2)°	*T* = 298 K
*V* = 1921.4 (4) Å^3^	Block, colorless
*Z* = 4	0.27 × 0.26 × 0.26 mm

### 2-(4-Fluorophenyl)-3-phenyl-2,3-dihydro-4H-pyrido[3,2-e][1,3]thiazin-4-one toluene hemisolvate (1) Data collection

**Table:**

Bruker CCD area detector diffractometer	4590 independent reflections
Radiation source: fine-focus sealed tube	3093 reflections with *I* > 2σ(*I*)
Parallel-graphite monochromator	*R*_int_ = 0.026
Detector resolution: 8.34 pixels mm^-1^	θ_max_ = 28.3°, θ_min_ = 1.6°
phi and ω scans	*h* = −18→19
Absorption correction: multi-scan (SADABS; Bruker, 2001)	*k* = −11→11
*T*_min_ = 0.877, *T*_max_ = 0.9	*l* = −21→20
14808 measured reflections	

### 2-(4-Fluorophenyl)-3-phenyl-2,3-dihydro-4H-pyrido[3,2-e][1,3]thiazin-4-one toluene hemisolvate (1) Refinement

**Table:**

Refinement on *F*^2^	Primary atom site location: structure-invariant direct methods
Least-squares matrix: full	Secondary atom site location: difference Fourier map
*R*[*F*^2^ > 2σ(*F*^2^)] = 0.047	Hydrogen site location: inferred from neighbouring sites
*wR*(*F*^2^) = 0.151	H-atom parameters constrained
*S* = 1.01	*w* = 1/[σ^2^(*F*_o_^2^) + (0.0821*P*)^2^ + 0.1824*P*] where *P* = (*F*_o_^2^ + 2*F*_c_^2^)/3
4590 reflections	(Δ/σ)_max_ < 0.001
266 parameters	Δρ_max_ = 0.27 e Å^−^^3^
57 restraints	Δρ_min_ = −0.22 e Å^−^^3^

### 2-(4-Fluorophenyl)-3-phenyl-2,3-dihydro-4H-pyrido[3,2-e][1,3]thiazin-4-one toluene hemisolvate (1) Special details

**Table:**

Experimental. The data collection nominally covered a full sphere of reciprocal space by a combination of 4 sets of ω scans each set at different φ and/or 2θ angles and each scan (10 s exposure) covering -0.300° degrees in ω. The crystal to detector distance was 5.82 cm.
Geometry. All esds (except the esd in the dihedral angle between two l.s. planes) are estimated using the full covariance matrix. The cell esds are taken into account individually in the estimation of esds in distances, angles and torsion angles; correlations between esds in cell parameters are only used when they are defined by crystal symmetry. An approximate (isotropic) treatment of cell esds is used for estimating esds involving l.s. planes.
Refinement. Refinement of F^2^ against ALL reflections. The weighted R-factor wR and goodness of fit S are based on F^2^, conventional R-factors R are based on F, with F set to zero for negative F^2^. The threshold expression of F^2^ > 2sigma(F^2^) is used only for calculating R-factors(gt) etc. and is not relevant to the choice of reflections for refinement. R-factors based on F^2^ are statistically about twice as large as those based on F, and R- factors based on ALL data will be even larger.

### 2-(4-Fluorophenyl)-3-phenyl-2,3-dihydro-4H-pyrido[3,2-e][1,3]thiazin-4-one toluene hemisolvate (1) Fractional atomic coordinates and isotropic or equivalent isotropic displacement parameters (Å^2^)

**Table:**

	*x*	*y*	*z*	*U*_iso_*/*U*_eq_	Occ. (<1)
C1	0.29551 (12)	0.63383 (19)	0.57705 (12)	0.0532 (4)	
H1	0.3212	0.5451	0.5589	0.064*	
C2	0.15230 (13)	0.8065 (2)	0.49787 (11)	0.0556 (4)	
C3	0.21206 (12)	0.92059 (18)	0.56667 (11)	0.0507 (4)	
C4	0.31888 (13)	0.9229 (2)	0.61149 (12)	0.0547 (4)	
C5	0.32028 (19)	1.1473 (2)	0.67453 (17)	0.0801 (6)	
H5	0.3569	1.2267	0.7100	0.096*	
C6	0.21563 (18)	1.1539 (2)	0.63701 (15)	0.0706 (5)	
H6	0.1823	1.2334	0.6490	0.085*	
C7	0.16061 (14)	1.0401 (2)	0.58099 (12)	0.0589 (4)	
H7	0.0894	1.0435	0.5529	0.071*	
C8	0.28175 (12)	0.59127 (17)	0.66097 (11)	0.0504 (4)	
C9	0.36712 (13)	0.5528 (2)	0.73966 (13)	0.0624 (5)	
H9	0.4317	0.5558	0.7402	0.075*	
C10	0.35817 (17)	0.5105 (2)	0.81643 (14)	0.0739 (6)	
H10	0.4158	0.4854	0.8688	0.089*	
C11	0.26220 (19)	0.5060 (2)	0.81421 (15)	0.0738 (6)	
C12	0.17612 (16)	0.5385 (2)	0.73843 (16)	0.0733 (6)	
H12	0.1118	0.5319	0.7383	0.088*	
C13	0.18591 (13)	0.5816 (2)	0.66148 (14)	0.0613 (5)	
H13	0.1275	0.6046	0.6092	0.074*	
C14	0.15587 (12)	0.5691 (2)	0.42719 (11)	0.0550 (4)	
C15	0.13424 (15)	0.6091 (2)	0.33997 (13)	0.0666 (5)	
H15	0.1450	0.7063	0.3270	0.080*	
C16	0.09640 (17)	0.5050 (3)	0.27128 (14)	0.0786 (6)	
H16	0.0807	0.5330	0.2120	0.094*	
C17	0.08185 (15)	0.3616 (3)	0.28964 (16)	0.0788 (6)	
H17	0.0575	0.2918	0.2433	0.095*	
C18	0.10316 (18)	0.3212 (3)	0.37627 (17)	0.0835 (6)	
H18	0.0932	0.2235	0.3890	0.100*	
C19	0.13963 (17)	0.4250 (2)	0.44561 (14)	0.0748 (6)	
H19	0.1531	0.3972	0.5045	0.090*	
F1	0.25250 (13)	0.46699 (18)	0.89080 (11)	0.1126 (5)	
N1	0.20053 (10)	0.67498 (16)	0.49958 (9)	0.0557 (4)	
N2	0.37270 (13)	1.03462 (19)	0.66340 (13)	0.0728 (5)	
O1	0.06489 (10)	0.83185 (16)	0.44210 (9)	0.0744 (4)	
S1	0.39249 (3)	0.77650 (6)	0.60028 (4)	0.06511 (19)	
C1A	0.4323 (6)	0.2876 (5)	0.5316 (6)	0.246 (7)	0.50
H1AA	0.4597	0.2762	0.5962	0.369*	0.50
H1AB	0.4497	0.2024	0.5059	0.369*	0.50
H1AC	0.3591	0.2969	0.5067	0.369*	0.50
C1B	0.4768 (3)	0.4249 (4)	0.5097 (4)	0.0998 (16)	0.50
C1C	0.5654 (3)	0.4916 (6)	0.5751 (3)	0.113 (4)	0.50
H1C	0.5980	0.4492	0.6324	0.136*	0.50
C1D	0.6056 (4)	0.6207 (6)	0.5558 (5)	0.136 (3)	0.50
H1D	0.6644	0.6636	0.6000	0.163*	0.50
C1E	0.5576 (6)	0.6857 (4)	0.4700 (5)	0.161 (4)	0.50
H1E	0.5842	0.7718	0.4570	0.193*	0.50
C1F	0.4695 (5)	0.6202 (6)	0.4041 (4)	0.158 (4)	0.50
H1F	0.4373	0.6627	0.3468	0.190*	0.50
C1G	0.4298 (4)	0.4911 (6)	0.4239 (3)	0.136 (5)	0.50
H1G	0.3711	0.4484	0.3795	0.163*	0.50

### 2-(4-Fluorophenyl)-3-phenyl-2,3-dihydro-4H-pyrido[3,2-e][1,3]thiazin-4-one toluene hemisolvate (1) Atomic displacement parameters (Å^2^)

**Table:**

	*U*^11^	*U*^22^	*U*^33^	*U*^12^	*U*^13^	*U*^23^
C1	0.0436 (8)	0.0512 (10)	0.0595 (10)	0.0101 (7)	0.0170 (7)	−0.0053 (8)
C2	0.0517 (9)	0.0555 (10)	0.0561 (10)	0.0126 (8)	0.0199 (8)	0.0024 (8)
C3	0.0547 (9)	0.0457 (9)	0.0540 (9)	0.0081 (7)	0.0255 (7)	0.0040 (7)
C4	0.0544 (9)	0.0493 (10)	0.0654 (10)	−0.0003 (7)	0.0305 (8)	0.0016 (8)
C5	0.0924 (16)	0.0543 (12)	0.0949 (16)	−0.0173 (11)	0.0413 (13)	−0.0139 (11)
C6	0.0978 (15)	0.0452 (11)	0.0830 (13)	0.0065 (10)	0.0523 (12)	−0.0023 (9)
C7	0.0667 (11)	0.0530 (10)	0.0640 (11)	0.0125 (8)	0.0347 (9)	0.0074 (8)
C8	0.0455 (8)	0.0399 (9)	0.0597 (9)	0.0051 (6)	0.0169 (7)	−0.0067 (7)
C9	0.0474 (9)	0.0641 (12)	0.0662 (11)	0.0045 (8)	0.0152 (8)	−0.0017 (9)
C10	0.0749 (13)	0.0709 (13)	0.0617 (12)	0.0033 (10)	0.0159 (10)	0.0035 (10)
C11	0.1004 (16)	0.0569 (12)	0.0735 (13)	0.0014 (11)	0.0463 (13)	−0.0014 (9)
C12	0.0716 (13)	0.0608 (13)	0.1025 (16)	0.0028 (10)	0.0515 (12)	−0.0017 (11)
C13	0.0476 (9)	0.0563 (11)	0.0754 (12)	0.0080 (8)	0.0221 (8)	−0.0024 (9)
C14	0.0494 (9)	0.0543 (10)	0.0550 (10)	0.0088 (7)	0.0166 (7)	−0.0053 (8)
C15	0.0721 (12)	0.0670 (12)	0.0653 (12)	0.0066 (9)	0.0339 (10)	−0.0018 (9)
C16	0.0800 (14)	0.0959 (18)	0.0593 (12)	0.0108 (12)	0.0295 (10)	−0.0122 (11)
C17	0.0637 (12)	0.0876 (17)	0.0733 (14)	0.0048 (11)	0.0183 (10)	−0.0297 (12)
C18	0.0901 (16)	0.0613 (13)	0.0875 (16)	−0.0057 (11)	0.0271 (12)	−0.0150 (11)
C19	0.0892 (14)	0.0607 (13)	0.0625 (12)	0.0023 (10)	0.0212 (11)	−0.0022 (9)
F1	0.1540 (14)	0.1109 (12)	0.0960 (10)	−0.0009 (10)	0.0757 (10)	0.0084 (8)
N1	0.0519 (7)	0.0490 (8)	0.0560 (8)	0.0110 (6)	0.0135 (6)	−0.0051 (6)
N2	0.0672 (10)	0.0594 (10)	0.0908 (12)	−0.0135 (8)	0.0331 (9)	−0.0106 (9)
O1	0.0562 (7)	0.0767 (9)	0.0685 (8)	0.0241 (6)	0.0063 (6)	−0.0078 (7)
S1	0.0459 (3)	0.0666 (3)	0.0863 (4)	0.0046 (2)	0.0318 (2)	−0.0013 (2)
C1A	0.304 (18)	0.126 (9)	0.42 (2)	−0.023 (10)	0.256 (17)	0.003 (11)
C1B	0.121 (4)	0.067 (3)	0.156 (5)	0.026 (3)	0.102 (4)	−0.001 (3)
C1C	0.128 (10)	0.109 (7)	0.124 (6)	0.021 (6)	0.075 (5)	−0.025 (5)
C1D	0.126 (5)	0.107 (5)	0.228 (8)	−0.018 (4)	0.128 (6)	−0.053 (5)
C1E	0.259 (12)	0.059 (4)	0.296 (11)	0.024 (5)	0.244 (10)	0.012 (6)
C1F	0.280 (11)	0.092 (5)	0.167 (6)	0.087 (6)	0.157 (6)	0.045 (4)
C1G	0.127 (10)	0.130 (9)	0.151 (8)	0.061 (6)	0.060 (6)	−0.004 (6)

### 2-(4-Fluorophenyl)-3-phenyl-2,3-dihydro-4H-pyrido[3,2-e][1,3]thiazin-4-one toluene hemisolvate (1) Geometric parameters (Å, º)

**Table:**

C1—H1	0.9800	C14—C15	1.370 (3)
C1—C8	1.517 (2)	C14—C19	1.376 (3)
C1—N1	1.459 (2)	C14—N1	1.439 (2)
C1—S1	1.8176 (18)	C15—H15	0.9300
C2—C3	1.494 (2)	C15—C16	1.383 (3)
C2—N1	1.370 (2)	C16—H16	0.9300
C2—O1	1.219 (2)	C16—C17	1.363 (3)
C3—C4	1.395 (2)	C17—H17	0.9300
C3—C7	1.385 (2)	C17—C18	1.363 (3)
C4—N2	1.331 (2)	C18—H18	0.9300
C4—S1	1.7534 (18)	C18—C19	1.387 (3)
C5—H5	0.9300	C19—H19	0.9300
C5—C6	1.367 (3)	C1A—H1AA	0.9600
C5—N2	1.326 (3)	C1A—H1AB	0.9600
C6—H6	0.9300	C1A—H1AC	0.9600
C6—C7	1.377 (3)	C1A—C1B	1.5071
C7—H7	0.9300	C1B—C1C	1.4021
C8—C9	1.390 (2)	C1B—C1G	1.4020
C8—C13	1.391 (2)	C1C—H1C	0.9300
C9—H9	0.9300	C1C—C1D	1.3962
C9—C10	1.370 (3)	C1D—H1D	0.9300
C10—H10	0.9300	C1D—C1E	1.3965
C10—C11	1.372 (3)	C1E—H1E	0.9300
C11—C12	1.357 (3)	C1E—C1F	1.3964
C11—F1	1.364 (2)	C1F—H1F	0.9300
C12—H12	0.9300	C1F—C1G	1.3964
C12—C13	1.381 (3)	C1G—H1G	0.9300
C13—H13	0.9300		
			
C8—C1—H1	106.4	C15—C14—C19	119.51 (17)
C8—C1—S1	112.28 (11)	C15—C14—N1	120.08 (17)
N1—C1—H1	106.4	C19—C14—N1	120.32 (16)
N1—C1—C8	114.11 (13)	C14—C15—H15	120.0
N1—C1—S1	110.74 (12)	C14—C15—C16	119.9 (2)
S1—C1—H1	106.4	C16—C15—H15	120.0
N1—C2—C3	117.14 (14)	C15—C16—H16	119.7
O1—C2—C3	120.75 (15)	C17—C16—C15	120.6 (2)
O1—C2—N1	122.10 (16)	C17—C16—H16	119.7
C4—C3—C2	123.80 (15)	C16—C17—H17	120.2
C7—C3—C2	118.96 (15)	C18—C17—C16	119.6 (2)
C7—C3—C4	116.90 (16)	C18—C17—H17	120.2
C3—C4—S1	121.16 (13)	C17—C18—H18	119.8
N2—C4—C3	123.87 (17)	C17—C18—C19	120.4 (2)
N2—C4—S1	114.95 (14)	C19—C18—H18	119.8
C6—C5—H5	118.0	C14—C19—C18	119.9 (2)
N2—C5—H5	118.0	C14—C19—H19	120.0
N2—C5—C6	123.96 (19)	C18—C19—H19	120.0
C5—C6—H6	120.8	C2—N1—C1	121.21 (14)
C5—C6—C7	118.49 (19)	C2—N1—C14	120.90 (14)
C7—C6—H6	120.8	C14—N1—C1	117.80 (13)
C3—C7—H7	120.2	C5—N2—C4	117.11 (18)
C6—C7—C3	119.57 (18)	C4—S1—C1	96.13 (8)
C6—C7—H7	120.2	C1C—C1B—C1A	120.9
C9—C8—C1	119.36 (15)	C1G—C1B—C1A	121.0
C9—C8—C13	117.94 (17)	C1G—C1B—C1C	118.1
C13—C8—C1	122.62 (15)	C1B—C1C—H1C	119.4
C8—C9—H9	119.3	C1D—C1C—C1B	121.1
C10—C9—C8	121.41 (18)	C1D—C1C—H1C	119.4
C10—C9—H9	119.3	C1C—C1D—H1D	119.9
C9—C10—H10	120.8	C1C—C1D—C1E	120.1
C9—C10—C11	118.47 (19)	C1E—C1D—H1D	119.9
C11—C10—H10	120.8	C1D—C1E—H1E	120.3
C12—C11—C10	122.5 (2)	C1F—C1E—C1D	119.4
C12—C11—F1	118.5 (2)	C1F—C1E—H1E	120.3
F1—C11—C10	119.0 (2)	C1E—C1F—H1F	119.9
C11—C12—H12	120.7	C1E—C1F—C1G	120.1
C11—C12—C13	118.65 (19)	C1G—C1F—H1F	119.9
C13—C12—H12	120.7	C1B—C1G—H1G	119.5
C8—C13—H13	119.5	C1F—C1G—C1B	121.1
C12—C13—C8	121.02 (18)	C1F—C1G—H1G	119.5
C12—C13—H13	119.5		
			
C1—C8—C9—C10	−178.56 (17)	C19—C14—C15—C16	0.0 (3)
C1—C8—C13—C12	178.16 (17)	C19—C14—N1—C1	51.1 (2)
C2—C3—C4—N2	170.57 (18)	C19—C14—N1—C2	−125.4 (2)
C2—C3—C4—S1	−7.9 (2)	F1—C11—C12—C13	178.40 (18)
C2—C3—C7—C6	−173.19 (17)	N1—C1—C8—C9	−178.73 (15)
C3—C2—N1—C1	14.2 (2)	N1—C1—C8—C13	4.6 (2)
C3—C2—N1—C14	−169.48 (15)	N1—C1—S1—C4	55.64 (13)
C3—C4—N2—C5	2.1 (3)	N1—C2—C3—C4	21.2 (3)
C3—C4—S1—C1	−27.46 (16)	N1—C2—C3—C7	−165.76 (16)
C4—C3—C7—C6	0.4 (2)	N1—C14—C15—C16	176.57 (17)
C5—C6—C7—C3	2.2 (3)	N1—C14—C19—C18	−175.56 (19)
C6—C5—N2—C4	0.8 (3)	N2—C4—S1—C1	153.98 (15)
C7—C3—C4—N2	−2.6 (3)	N2—C5—C6—C7	−3.0 (3)
C7—C3—C4—S1	178.93 (13)	O1—C2—C3—C4	−157.79 (18)
C8—C1—N1—C2	72.3 (2)	O1—C2—C3—C7	15.3 (3)
C8—C1—N1—C14	−104.19 (17)	O1—C2—N1—C1	−166.91 (17)
C8—C1—S1—C4	−73.21 (12)	O1—C2—N1—C14	9.4 (3)
C8—C9—C10—C11	0.3 (3)	S1—C1—C8—C9	−51.67 (19)
C9—C8—C13—C12	1.5 (3)	S1—C1—C8—C13	131.69 (15)
C9—C10—C11—C12	1.6 (3)	S1—C1—N1—C2	−55.58 (19)
C9—C10—C11—F1	−178.67 (19)	S1—C1—N1—C14	127.96 (14)
C10—C11—C12—C13	−1.8 (3)	S1—C4—N2—C5	−179.41 (16)
C11—C12—C13—C8	0.3 (3)	C1A—C1B—C1C—C1D	−178.5
C13—C8—C9—C10	−1.8 (3)	C1A—C1B—C1G—C1F	178.5
C14—C15—C16—C17	−1.1 (3)	C1B—C1C—C1D—C1E	−0.1
C15—C14—C19—C18	1.0 (3)	C1C—C1B—C1G—C1F	−0.3
C15—C14—N1—C1	−125.51 (18)	C1C—C1D—C1E—C1F	−0.1
C15—C14—N1—C2	58.0 (2)	C1D—C1E—C1F—C1G	0.1
C15—C16—C17—C18	1.1 (3)	C1E—C1F—C1G—C1B	0.1
C16—C17—C18—C19	−0.1 (3)	C1G—C1B—C1C—C1D	0.3
C17—C18—C19—C14	−1.0 (3)		

### 2-(4-Fluorophenyl)-3-phenyl-2,3-dihydro-4H-pyrido[3,2-e][1,3]thiazin-4-one toluene hemisolvate (1) Hydrogen-bond geometry (Å, º)

**Table:**

*D*—H···*A*	*D*—H	H···*A*	*D*···*A*	*D*—H···*A*
C7—H7···O1^i^	0.93	2.53	3.321 (2)	143
C9—H9···N2^ii^	0.93	2.58	3.398 (2)	147

Symmetry codes: (i) −*x*, −*y*+2, −*z*+1; (ii) −*x*+1, *y*−1/2, −*z*+3/2.

### 2-(4-Nitrophenyl)-3-phenyl-2,3-dihydro-4H-pyrido[3,2-e][1,3]thiazin-4-one isopropanol 0.25-solvate 0.0625-hydrate (2) Crystal data

**Table:**

4C_19_H_13_N_3_O_3_S·C_3_H_8_O·0.25H_2_O	*Z* = 2
*M**_r_* = 1518.13	*F*(000) = 1577
Triclinic, *P*1	*D*_x_ = 1.412 Mg m^−^^3^
*a* = 12.5451 (13) Å	Mo *K*α radiation, λ = 0.71073 Å
*b* = 15.9804 (17) Å	Cell parameters from 4843 reflections
*c* = 19.434 (2) Å	θ = 2.4–28.2°
α = 86.671 (2)°	µ = 0.21 mm^−^^1^
β = 72.369 (2)°	*T* = 298 K
γ = 74.167 (2)°	Plate, colorless
*V* = 3570.8 (6) Å^3^	0.19 × 0.18 × 0.03 mm

### 2-(4-Nitrophenyl)-3-phenyl-2,3-dihydro-4H-pyrido[3,2-e][1,3]thiazin-4-one isopropanol 0.25-solvate 0.0625-hydrate (2) Data collection

**Table:**

Bruker CCD area detector diffractometer	7969 reflections with *I* > 2σ(*I*)
phi and ω scans	*R*_int_ = 0.039
Absorption correction: multi-scan (SADABS; Bruker, 2001)	θ_max_ = 28.4°, θ_min_ = 1.3°
*T*_min_ = 0.230, *T*_max_ = 0.9	*h* = −16→16
30898 measured reflections	*k* = −20→20
16372 independent reflections	*l* = −25→25

### 2-(4-Nitrophenyl)-3-phenyl-2,3-dihydro-4H-pyrido[3,2-e][1,3]thiazin-4-one isopropanol 0.25-solvate 0.0625-hydrate (2) Refinement

**Table:**

Refinement on *F*^2^	30 restraints
Least-squares matrix: full	Hydrogen site location: mixed
*R*[*F*^2^ > 2σ(*F*^2^)] = 0.057	H-atom parameters constrained
*wR*(*F*^2^) = 0.158	*w* = 1/[σ^2^(*F*_o_^2^) + (0.0674*P*)^2^] where *P* = (*F*_o_^2^ + 2*F*_c_^2^)/3
*S* = 0.95	(Δ/σ)_max_ = 0.002
16372 reflections	Δρ_max_ = 0.28 e Å^−^^3^
997 parameters	Δρ_min_ = −0.27 e Å^−^^3^

### 2-(4-Nitrophenyl)-3-phenyl-2,3-dihydro-4H-pyrido[3,2-e][1,3]thiazin-4-one isopropanol 0.25-solvate 0.0625-hydrate (2) Special details

**Table:**

Experimental. The data collection nominally covered a full sphere of reciprocal space by a combination of 4 sets of ω scans each set at different φ and/or 2θ angles and each scan (10 s exposure) covering -0.300° degrees in ω. The crystal to detector distance was 5.82 cm.
Geometry. All esds (except the esd in the dihedral angle between two l.s. planes) are estimated using the full covariance matrix. The cell esds are taken into account individually in the estimation of esds in distances, angles and torsion angles; correlations between esds in cell parameters are only used when they are defined by crystal symmetry. An approximate (isotropic) treatment of cell esds is used for estimating esds involving l.s. planes.

### 2-(4-Nitrophenyl)-3-phenyl-2,3-dihydro-4H-pyrido[3,2-e][1,3]thiazin-4-one isopropanol 0.25-solvate 0.0625-hydrate (2) Fractional atomic coordinates and isotropic or equivalent isotropic displacement parameters (Å^2^)

**Table:**

	*x*	*y*	*z*	*U*_iso_*/*U*_eq_	Occ. (<1)
S1	0.29218 (6)	0.03290 (5)	0.18613 (4)	0.0501 (2)	
O1	−0.04665 (16)	0.18932 (12)	0.17939 (10)	0.0550 (5)	
O2	−0.0412 (3)	−0.27498 (17)	0.35233 (15)	0.0955 (8)	
O3	0.0788 (3)	−0.29499 (16)	0.41328 (14)	0.0907 (8)	
N1	0.06953 (17)	0.12964 (14)	0.24885 (11)	0.0414 (5)	
N2	0.2998 (2)	−0.01955 (15)	0.05960 (13)	0.0535 (6)	
N3	0.0313 (3)	−0.25406 (18)	0.37145 (15)	0.0632 (7)	
C1	0.1603 (2)	0.05663 (17)	0.26243 (14)	0.0437 (7)	
H1	0.181510	0.076401	0.302031	0.052*	
C2	0.0381 (2)	0.13447 (18)	0.18683 (14)	0.0426 (7)	
C3	0.1134 (2)	0.07162 (17)	0.12647 (14)	0.0415 (6)	
C4	0.2284 (2)	0.02717 (18)	0.11932 (14)	0.0440 (7)	
C5	0.2559 (3)	−0.0235 (2)	0.00611 (17)	0.0597 (8)	
H5	0.304997	−0.054121	−0.036014	0.072*	
C6	0.1422 (3)	0.0148 (2)	0.00931 (16)	0.0580 (8)	
H6	0.114623	0.007952	−0.028769	0.070*	
C7	0.0699 (3)	0.06374 (19)	0.07008 (15)	0.0502 (7)	
H7	−0.007133	0.091139	0.073294	0.060*	
C8	0.0037 (2)	0.19178 (19)	0.30682 (15)	0.0459 (7)	
C9	−0.0185 (2)	0.28007 (19)	0.29480 (17)	0.0504 (7)	
H9	0.008459	0.299760	0.248466	0.060*	
C10	−0.0806 (3)	0.3390 (2)	0.3512 (2)	0.0633 (9)	
H10	−0.096270	0.398356	0.342596	0.076*	
C11	−0.1193 (3)	0.3108 (3)	0.4201 (2)	0.0761 (11)	
H11	−0.161026	0.350794	0.458010	0.091*	
C12	−0.0963 (3)	0.2229 (3)	0.43276 (18)	0.0784 (11)	
H12	−0.121912	0.203669	0.479409	0.094*	
C13	−0.0352 (3)	0.1633 (2)	0.37638 (16)	0.0617 (9)	
H13	−0.020190	0.104029	0.385098	0.074*	
C14	0.1239 (2)	−0.02529 (17)	0.28802 (14)	0.0412 (6)	
C15	0.1940 (2)	−0.08729 (19)	0.32026 (15)	0.0518 (8)	
H15	0.262207	−0.077937	0.323669	0.062*	
C16	0.1648 (3)	−0.16179 (19)	0.34711 (15)	0.0532 (8)	
H16	0.212076	−0.202681	0.368797	0.064*	
C17	0.0643 (3)	−0.17473 (19)	0.34131 (14)	0.0479 (7)	
C18	−0.0068 (3)	−0.1158 (2)	0.30966 (15)	0.0532 (8)	
H18	−0.074127	−0.126398	0.305862	0.064*	
C19	0.0226 (2)	−0.04033 (19)	0.28338 (15)	0.0491 (7)	
H19	−0.025836	0.000607	0.262464	0.059*	
S2	0.19473 (6)	0.08666 (5)	0.80114 (5)	0.0613 (2)	
O4	0.56806 (17)	−0.00558 (13)	0.70493 (10)	0.0574 (5)	
O5	0.2168 (3)	0.25409 (17)	1.12494 (13)	0.0845 (7)	
O6	0.4024 (3)	0.20792 (19)	1.09163 (13)	0.0952 (8)	
N4	0.4121 (2)	0.10919 (15)	0.74362 (12)	0.0500 (6)	
N5	0.2221 (2)	−0.07471 (19)	0.83891 (14)	0.0596 (7)	
N6	0.3093 (3)	0.22173 (18)	1.08058 (15)	0.0633 (7)	
C20	0.3005 (2)	0.14686 (18)	0.79718 (15)	0.0498 (7)	
H20	0.269897	0.204631	0.779922	0.060*	
C21	0.4679 (2)	0.02283 (19)	0.74233 (15)	0.0460 (7)	
C22	0.4024 (2)	−0.03636 (18)	0.78632 (14)	0.0436 (7)	
C23	0.2810 (2)	−0.0166 (2)	0.81035 (15)	0.0496 (7)	
C24	0.2841 (3)	−0.1547 (2)	0.84727 (17)	0.0632 (9)	
H24	0.244423	−0.196368	0.865752	0.076*	
C25	0.4028 (3)	−0.1794 (2)	0.83030 (17)	0.0593 (8)	
H25	0.441822	−0.234882	0.840296	0.071*	
C26	0.4626 (3)	−0.12010 (19)	0.79817 (16)	0.0518 (7)	
H26	0.543281	−0.135961	0.784345	0.062*	
C27	0.4675 (2)	0.16600 (19)	0.69490 (16)	0.0511 (7)	
C28	0.4947 (3)	0.1525 (2)	0.62135 (17)	0.0714 (10)	
H28	0.477424	0.106687	0.603497	0.086*	
C29	0.5478 (3)	0.2075 (3)	0.5746 (2)	0.0849 (12)	
H29	0.567327	0.198167	0.525004	0.102*	
C30	0.5721 (3)	0.2757 (3)	0.6005 (2)	0.0811 (11)	
H30	0.607450	0.312659	0.568546	0.097*	
C31	0.5445 (3)	0.2897 (2)	0.6732 (2)	0.0740 (10)	
H31	0.560919	0.336212	0.690674	0.089*	
C32	0.4921 (3)	0.2349 (2)	0.72099 (18)	0.0605 (8)	
H32	0.473560	0.244328	0.770509	0.073*	
C33	0.3067 (2)	0.16168 (17)	0.87210 (15)	0.0433 (7)	
C34	0.4083 (2)	0.13472 (18)	0.89128 (16)	0.0507 (7)	
H34	0.476271	0.103994	0.857772	0.061*	
C35	0.4099 (3)	0.15283 (19)	0.95934 (16)	0.0544 (8)	
H35	0.478404	0.135045	0.971940	0.065*	
C36	0.3084 (3)	0.19765 (18)	1.00816 (15)	0.0479 (7)	
C37	0.2059 (3)	0.22324 (18)	0.99185 (16)	0.0518 (7)	
H37	0.137821	0.252247	1.026239	0.062*	
C38	0.2052 (2)	0.20535 (18)	0.92384 (16)	0.0493 (7)	
H38	0.135942	0.222643	0.912096	0.059*	
S3	0.48325 (6)	0.54203 (5)	0.79720 (5)	0.0604 (2)	
O7	0.13744 (16)	0.53956 (13)	0.79718 (11)	0.0584 (5)	
O8	0.2856 (3)	0.96125 (17)	0.64375 (14)	0.0961 (9)	
O9	0.4647 (3)	0.92989 (18)	0.58597 (18)	0.1164 (11)	
N7	0.32514 (18)	0.52725 (15)	0.73153 (12)	0.0476 (6)	
N8	0.3661 (2)	0.61800 (17)	0.92278 (15)	0.0650 (7)	
N9	0.3801 (3)	0.9097 (2)	0.62374 (15)	0.0657 (7)	
C39	0.4283 (2)	0.55876 (18)	0.71966 (16)	0.0494 (7)	
H39	0.488822	0.522418	0.680202	0.059*	
C40	0.2314 (2)	0.55268 (18)	0.79167 (16)	0.0479 (7)	
C41	0.2460 (2)	0.59634 (18)	0.85278 (15)	0.0467 (7)	
C42	0.3535 (2)	0.59082 (18)	0.86259 (16)	0.0518 (7)	
C43	0.2685 (4)	0.6551 (2)	0.97386 (19)	0.0726 (10)	
H43	0.274854	0.673113	1.016759	0.087*	
C44	0.1595 (3)	0.6684 (2)	0.96741 (18)	0.0682 (9)	
H44	0.094536	0.697668	1.003856	0.082*	
C45	0.1474 (3)	0.63801 (19)	0.90639 (16)	0.0559 (8)	
H45	0.074191	0.645318	0.901185	0.067*	
C46	0.3174 (2)	0.4816 (2)	0.67217 (17)	0.0519 (8)	
C47	0.3583 (3)	0.5075 (2)	0.60260 (19)	0.0687 (9)	
H47	0.390137	0.554651	0.593770	0.082*	
C48	0.3516 (3)	0.4627 (3)	0.5459 (2)	0.0886 (12)	
H48	0.380530	0.479097	0.498715	0.106*	
C49	0.3020 (3)	0.3938 (3)	0.5591 (3)	0.0927 (13)	
H49	0.295729	0.364785	0.521042	0.111*	
C50	0.2623 (3)	0.3685 (3)	0.6286 (3)	0.0845 (12)	
H50	0.229480	0.321849	0.637564	0.101*	
C51	0.2705 (3)	0.4115 (2)	0.6853 (2)	0.0646 (9)	
H51	0.244613	0.393393	0.732351	0.078*	
C52	0.4134 (2)	0.65204 (18)	0.69454 (14)	0.0442 (7)	
C53	0.3052 (2)	0.71039 (19)	0.70482 (15)	0.0499 (7)	
H53	0.238603	0.692619	0.727595	0.060*	
C54	0.2952 (2)	0.79473 (19)	0.68155 (15)	0.0509 (7)	
H54	0.222522	0.834073	0.689347	0.061*	
C55	0.3930 (3)	0.81948 (19)	0.64715 (15)	0.0492 (7)	
C56	0.5027 (3)	0.7632 (2)	0.63555 (16)	0.0572 (8)	
H56	0.568763	0.781216	0.612004	0.069*	
C57	0.5112 (2)	0.6801 (2)	0.65971 (16)	0.0554 (8)	
H57	0.584312	0.641513	0.652624	0.066*	
S4	−0.00824 (6)	0.62257 (5)	0.19021 (5)	0.0601 (2)	
O10	0.27502 (19)	0.58431 (14)	0.28290 (11)	0.0660 (6)	
O11	0.4765 (3)	0.3508 (2)	−0.09779 (15)	0.1215 (11)	
O12	0.3228 (3)	0.37649 (17)	−0.13080 (14)	0.0992 (9)	
N10	0.1560 (2)	0.52364 (15)	0.24786 (12)	0.0486 (6)	
N11	0.0602 (2)	0.76000 (18)	0.14094 (15)	0.0687 (8)	
N12	0.3728 (3)	0.37844 (19)	−0.08640 (17)	0.0780 (9)	
C58	0.0938 (2)	0.51872 (18)	0.19627 (15)	0.0481 (7)	
H58	0.047299	0.477654	0.215668	0.058*	
C59	0.2114 (3)	0.5869 (2)	0.24570 (16)	0.0506 (7)	
C60	0.1878 (2)	0.66125 (19)	0.19797 (15)	0.0479 (7)	
C61	0.0896 (2)	0.68606 (19)	0.17441 (16)	0.0537 (8)	
C62	0.1314 (4)	0.8109 (2)	0.1284 (2)	0.0767 (10)	
H62	0.110636	0.863692	0.106534	0.092*	
C63	0.2332 (3)	0.7909 (2)	0.14539 (18)	0.0709 (10)	
H63	0.282649	0.826976	0.132484	0.085*	
C64	0.2609 (3)	0.7155 (2)	0.18231 (16)	0.0585 (8)	
H64	0.327943	0.701254	0.196506	0.070*	
C65	0.1627 (3)	0.4563 (2)	0.30032 (17)	0.0534 (8)	
C66	0.1160 (3)	0.4788 (2)	0.37276 (18)	0.0774 (10)	
H66	0.082455	0.536872	0.387554	0.093*	
C67	0.1197 (4)	0.4145 (3)	0.4230 (2)	0.0930 (12)	
H67	0.089317	0.429214	0.472005	0.112*	
C68	0.1681 (3)	0.3289 (3)	0.4009 (2)	0.0830 (12)	
H68	0.168995	0.285587	0.435030	0.100*	
C69	0.2145 (3)	0.3072 (2)	0.3297 (2)	0.0749 (10)	
H69	0.248019	0.249089	0.315232	0.090*	
C70	0.2125 (3)	0.3707 (2)	0.27805 (18)	0.0606 (8)	
H70	0.244378	0.355613	0.229149	0.073*	
C71	0.1711 (2)	0.48386 (17)	0.12155 (14)	0.0425 (7)	
C72	0.2909 (2)	0.46199 (19)	0.10238 (17)	0.0545 (8)	
H72	0.327051	0.470172	0.135774	0.065*	
C73	0.3576 (3)	0.4282 (2)	0.03438 (18)	0.0608 (8)	
H73	0.438223	0.413308	0.021859	0.073*	
C74	0.3033 (3)	0.41698 (19)	−0.01417 (16)	0.0561 (8)	
C75	0.1842 (3)	0.43826 (19)	0.00298 (17)	0.0591 (8)	
H75	0.148616	0.430409	−0.030836	0.071*	
C76	0.1192 (3)	0.47116 (18)	0.07068 (16)	0.0510 (7)	
H76	0.038687	0.485333	0.082884	0.061*	
O13	0.2504 (2)	0.10492 (18)	0.40343 (12)	0.1046 (9)	
H13A	0.303804	0.061308	0.387928	0.157*	0.58 (2)
H13B	0.302801	0.076905	0.369810	0.157*	0.42 (2)
C77	0.1668 (5)	0.0177 (4)	0.4961 (3)	0.1325 (18)	
H77A	0.109954	0.014164	0.473447	0.199*	0.58 (2)
H77B	0.135100	0.015458	0.547577	0.199*	0.58 (2)
H77C	0.234562	−0.030317	0.479476	0.199*	0.58 (2)
H77D	0.092788	0.060158	0.507324	0.199*	0.42 (2)
H77E	0.171687	−0.017491	0.537416	0.199*	0.42 (2)
H77F	0.175255	−0.018666	0.456135	0.199*	0.42 (2)
C79	0.2605 (4)	0.1265 (3)	0.5221 (2)	0.1003 (13)	
H79A	0.338032	0.089162	0.511710	0.150*	0.58 (2)
H79B	0.220463	0.124443	0.572520	0.150*	0.58 (2)
H79C	0.263384	0.185085	0.509982	0.150*	0.58 (2)
H79D	0.324091	0.151322	0.500445	0.150*	0.42 (2)
H79E	0.267326	0.101210	0.567084	0.150*	0.42 (2)
H79F	0.188591	0.171108	0.530563	0.150*	0.42 (2)
O14	0.3199 (9)	0.5360 (8)	0.4020 (6)	0.099 (3)	0.25
H14A	0.283502	0.500854	0.426031	0.148*	0.25
H14B	0.277331	0.587867	0.411647	0.148*	0.25
C78A	0.1974 (14)	0.0965 (10)	0.4784 (4)	0.077 (3)	0.58 (2)
H78A	0.122575	0.140645	0.488887	0.092*	0.58 (2)
C78B	0.2625 (18)	0.0633 (10)	0.4765 (6)	0.079 (4)	0.42 (2)
H78B	0.337343	0.019326	0.466878	0.095*	0.42 (2)

### 2-(4-Nitrophenyl)-3-phenyl-2,3-dihydro-4H-pyrido[3,2-e][1,3]thiazin-4-one isopropanol 0.25-solvate 0.0625-hydrate (2) Atomic displacement parameters (Å^2^)

**Table:**

	*U*^11^	*U*^22^	*U*^33^	*U*^12^	*U*^13^	*U*^23^
S1	0.0371 (4)	0.0596 (5)	0.0533 (5)	−0.0121 (3)	−0.0145 (3)	0.0032 (4)
O1	0.0471 (11)	0.0562 (13)	0.0612 (13)	−0.0020 (10)	−0.0250 (10)	−0.0045 (10)
O2	0.118 (2)	0.092 (2)	0.104 (2)	−0.0665 (18)	−0.0394 (18)	0.0080 (16)
O3	0.134 (2)	0.0693 (17)	0.0782 (18)	−0.0354 (16)	−0.0412 (17)	0.0227 (14)
N1	0.0405 (12)	0.0433 (13)	0.0402 (13)	−0.0069 (10)	−0.0150 (10)	−0.0031 (11)
N2	0.0485 (14)	0.0568 (16)	0.0490 (15)	−0.0128 (12)	−0.0063 (12)	−0.0020 (13)
N3	0.085 (2)	0.0573 (18)	0.0483 (17)	−0.0295 (16)	−0.0111 (15)	−0.0055 (14)
C1	0.0409 (15)	0.0486 (17)	0.0448 (16)	−0.0104 (13)	−0.0186 (13)	0.0010 (13)
C2	0.0393 (15)	0.0488 (17)	0.0439 (17)	−0.0152 (13)	−0.0152 (13)	0.0003 (14)
C3	0.0432 (15)	0.0438 (16)	0.0396 (16)	−0.0157 (13)	−0.0117 (13)	0.0010 (13)
C4	0.0409 (15)	0.0452 (17)	0.0428 (16)	−0.0136 (13)	−0.0063 (13)	0.0031 (14)
C5	0.062 (2)	0.067 (2)	0.0471 (19)	−0.0188 (17)	−0.0077 (16)	−0.0118 (16)
C6	0.065 (2)	0.070 (2)	0.0433 (18)	−0.0245 (17)	−0.0152 (16)	−0.0056 (16)
C7	0.0505 (17)	0.0538 (18)	0.0510 (18)	−0.0192 (14)	−0.0174 (15)	0.0014 (15)
C8	0.0388 (15)	0.0519 (19)	0.0466 (18)	−0.0087 (13)	−0.0141 (13)	−0.0063 (15)
C9	0.0445 (16)	0.0509 (19)	0.0591 (19)	−0.0116 (14)	−0.0201 (15)	−0.0054 (16)
C10	0.0559 (19)	0.056 (2)	0.076 (2)	−0.0039 (16)	−0.0243 (18)	−0.0153 (19)
C11	0.065 (2)	0.081 (3)	0.072 (3)	0.006 (2)	−0.022 (2)	−0.032 (2)
C12	0.082 (3)	0.089 (3)	0.048 (2)	−0.001 (2)	−0.0141 (18)	−0.012 (2)
C13	0.071 (2)	0.058 (2)	0.050 (2)	−0.0072 (17)	−0.0170 (17)	−0.0056 (17)
C14	0.0417 (15)	0.0468 (17)	0.0373 (15)	−0.0126 (13)	−0.0136 (12)	−0.0019 (13)
C15	0.0451 (16)	0.058 (2)	0.0575 (19)	−0.0149 (14)	−0.0222 (14)	0.0071 (16)
C16	0.0523 (18)	0.0507 (19)	0.0534 (19)	−0.0086 (15)	−0.0171 (15)	0.0070 (15)
C17	0.0583 (18)	0.0483 (18)	0.0366 (16)	−0.0187 (15)	−0.0089 (14)	−0.0022 (14)
C18	0.0544 (18)	0.067 (2)	0.0487 (18)	−0.0285 (16)	−0.0204 (15)	0.0026 (16)
C19	0.0484 (17)	0.0560 (19)	0.0491 (17)	−0.0175 (14)	−0.0215 (14)	0.0068 (15)
S2	0.0445 (4)	0.0671 (5)	0.0745 (6)	−0.0002 (4)	−0.0304 (4)	−0.0187 (4)
O4	0.0454 (12)	0.0598 (13)	0.0554 (12)	−0.0021 (10)	−0.0076 (10)	−0.0059 (10)
O5	0.109 (2)	0.0879 (19)	0.0523 (15)	−0.0279 (16)	−0.0143 (15)	−0.0117 (13)
O6	0.105 (2)	0.126 (2)	0.0771 (18)	−0.0361 (18)	−0.0556 (16)	0.0061 (16)
N4	0.0520 (14)	0.0469 (15)	0.0442 (14)	−0.0031 (12)	−0.0126 (12)	−0.0044 (12)
N5	0.0523 (15)	0.0680 (19)	0.0641 (17)	−0.0232 (15)	−0.0161 (13)	−0.0142 (15)
N6	0.090 (2)	0.0581 (18)	0.0512 (18)	−0.0301 (17)	−0.0269 (18)	0.0066 (14)
C20	0.0483 (17)	0.0461 (17)	0.0508 (18)	−0.0007 (13)	−0.0182 (14)	−0.0052 (14)
C21	0.0444 (16)	0.0495 (18)	0.0423 (17)	−0.0029 (14)	−0.0172 (14)	−0.0098 (14)
C22	0.0418 (15)	0.0507 (18)	0.0411 (16)	−0.0100 (14)	−0.0168 (13)	−0.0082 (14)
C23	0.0458 (17)	0.059 (2)	0.0476 (18)	−0.0087 (15)	−0.0201 (14)	−0.0175 (15)
C24	0.072 (2)	0.064 (2)	0.062 (2)	−0.0297 (19)	−0.0208 (18)	−0.0054 (18)
C25	0.067 (2)	0.055 (2)	0.064 (2)	−0.0179 (17)	−0.0284 (17)	0.0002 (17)
C26	0.0504 (17)	0.0536 (19)	0.0540 (18)	−0.0098 (15)	−0.0217 (15)	−0.0063 (15)
C27	0.0533 (17)	0.0461 (18)	0.0500 (19)	−0.0055 (14)	−0.0169 (15)	0.0009 (15)
C28	0.093 (3)	0.071 (2)	0.048 (2)	−0.016 (2)	−0.0235 (19)	0.0020 (18)
C29	0.102 (3)	0.089 (3)	0.055 (2)	−0.022 (2)	−0.018 (2)	0.014 (2)
C30	0.072 (2)	0.074 (3)	0.085 (3)	−0.013 (2)	−0.017 (2)	0.027 (2)
C31	0.066 (2)	0.058 (2)	0.099 (3)	−0.0140 (18)	−0.030 (2)	0.008 (2)
C32	0.066 (2)	0.054 (2)	0.060 (2)	−0.0117 (17)	−0.0209 (17)	0.0003 (17)
C33	0.0431 (15)	0.0356 (15)	0.0492 (17)	−0.0056 (12)	−0.0149 (13)	−0.0013 (13)
C34	0.0441 (16)	0.0520 (18)	0.0513 (19)	−0.0027 (14)	−0.0152 (14)	−0.0060 (15)
C35	0.0551 (19)	0.0558 (19)	0.058 (2)	−0.0120 (15)	−0.0289 (16)	0.0057 (16)
C36	0.0624 (19)	0.0414 (17)	0.0448 (17)	−0.0185 (15)	−0.0194 (15)	0.0020 (14)
C37	0.0518 (18)	0.0465 (18)	0.0534 (19)	−0.0116 (14)	−0.0101 (15)	−0.0068 (15)
C38	0.0413 (16)	0.0491 (18)	0.0584 (19)	−0.0082 (13)	−0.0181 (14)	−0.0063 (15)
S3	0.0437 (4)	0.0639 (5)	0.0757 (6)	−0.0115 (4)	−0.0240 (4)	0.0039 (4)
O7	0.0379 (11)	0.0709 (14)	0.0660 (14)	−0.0219 (10)	−0.0072 (10)	−0.0050 (11)
O8	0.103 (2)	0.0682 (17)	0.097 (2)	−0.0031 (16)	−0.0212 (17)	0.0161 (15)
O9	0.091 (2)	0.090 (2)	0.165 (3)	−0.0441 (17)	−0.024 (2)	0.052 (2)
N7	0.0384 (13)	0.0525 (15)	0.0515 (15)	−0.0166 (11)	−0.0075 (11)	−0.0059 (12)
N8	0.079 (2)	0.0635 (18)	0.0610 (18)	−0.0200 (15)	−0.0319 (16)	0.0020 (15)
N9	0.079 (2)	0.066 (2)	0.0620 (18)	−0.0295 (18)	−0.0288 (17)	0.0136 (16)
C39	0.0333 (14)	0.0513 (18)	0.0602 (19)	−0.0134 (13)	−0.0061 (13)	−0.0056 (15)
C40	0.0429 (17)	0.0461 (17)	0.0529 (18)	−0.0137 (14)	−0.0108 (14)	0.0042 (14)
C41	0.0462 (16)	0.0459 (17)	0.0497 (18)	−0.0166 (14)	−0.0139 (14)	0.0055 (14)
C42	0.0556 (18)	0.0455 (18)	0.057 (2)	−0.0149 (14)	−0.0217 (15)	0.0101 (15)
C43	0.096 (3)	0.068 (2)	0.058 (2)	−0.020 (2)	−0.028 (2)	−0.0015 (19)
C44	0.076 (2)	0.060 (2)	0.058 (2)	−0.0113 (18)	−0.0096 (18)	−0.0072 (17)
C45	0.0539 (18)	0.0546 (19)	0.054 (2)	−0.0118 (15)	−0.0107 (16)	0.0016 (16)
C46	0.0377 (15)	0.0528 (19)	0.062 (2)	−0.0078 (14)	−0.0131 (15)	−0.0095 (16)
C47	0.069 (2)	0.070 (2)	0.061 (2)	−0.0176 (18)	−0.0110 (18)	−0.0083 (19)
C48	0.090 (3)	0.104 (3)	0.064 (3)	−0.013 (3)	−0.018 (2)	−0.020 (2)
C49	0.081 (3)	0.111 (4)	0.095 (3)	−0.018 (3)	−0.038 (3)	−0.042 (3)
C50	0.066 (2)	0.087 (3)	0.109 (3)	−0.031 (2)	−0.022 (2)	−0.033 (3)
C51	0.0478 (18)	0.067 (2)	0.082 (2)	−0.0231 (16)	−0.0138 (17)	−0.0099 (19)
C52	0.0354 (15)	0.0520 (18)	0.0475 (17)	−0.0168 (13)	−0.0102 (13)	−0.0036 (14)
C53	0.0376 (15)	0.057 (2)	0.0585 (19)	−0.0199 (14)	−0.0130 (14)	0.0012 (16)
C54	0.0429 (16)	0.054 (2)	0.0564 (19)	−0.0126 (14)	−0.0167 (14)	0.0034 (15)
C55	0.0572 (19)	0.0515 (19)	0.0449 (17)	−0.0194 (15)	−0.0195 (15)	0.0027 (14)
C56	0.0469 (18)	0.064 (2)	0.064 (2)	−0.0277 (16)	−0.0099 (15)	0.0058 (17)
C57	0.0362 (15)	0.057 (2)	0.071 (2)	−0.0153 (14)	−0.0106 (15)	0.0020 (17)
S4	0.0411 (4)	0.0577 (5)	0.0746 (6)	−0.0084 (4)	−0.0097 (4)	−0.0085 (4)
O10	0.0748 (15)	0.0827 (16)	0.0578 (13)	−0.0422 (13)	−0.0262 (12)	0.0025 (12)
O11	0.095 (2)	0.132 (3)	0.086 (2)	0.011 (2)	0.0140 (18)	−0.0144 (18)
O12	0.151 (3)	0.0831 (19)	0.0562 (16)	−0.0310 (18)	−0.0192 (18)	−0.0104 (14)
N10	0.0553 (14)	0.0547 (15)	0.0427 (14)	−0.0257 (12)	−0.0150 (12)	0.0023 (12)
N11	0.0681 (18)	0.0474 (17)	0.079 (2)	−0.0047 (15)	−0.0136 (15)	−0.0069 (15)
N12	0.099 (3)	0.0574 (19)	0.057 (2)	−0.0146 (18)	0.000 (2)	0.0009 (16)
C58	0.0467 (16)	0.0489 (18)	0.0529 (18)	−0.0194 (14)	−0.0150 (14)	0.0003 (14)
C59	0.0500 (17)	0.057 (2)	0.0453 (18)	−0.0219 (15)	−0.0071 (14)	−0.0076 (15)
C60	0.0482 (17)	0.0467 (18)	0.0445 (17)	−0.0159 (14)	−0.0029 (14)	−0.0086 (14)
C61	0.0481 (17)	0.0453 (18)	0.0540 (19)	−0.0038 (14)	−0.0010 (15)	−0.0123 (15)
C62	0.089 (3)	0.049 (2)	0.079 (3)	−0.009 (2)	−0.014 (2)	0.0016 (19)
C63	0.085 (3)	0.052 (2)	0.067 (2)	−0.0310 (19)	0.001 (2)	−0.0010 (18)
C64	0.0565 (19)	0.060 (2)	0.0548 (19)	−0.0230 (16)	−0.0025 (15)	−0.0088 (17)
C65	0.0562 (18)	0.056 (2)	0.053 (2)	−0.0225 (16)	−0.0169 (15)	0.0050 (16)
C66	0.107 (3)	0.071 (2)	0.050 (2)	−0.028 (2)	−0.013 (2)	0.0068 (19)
C67	0.119 (3)	0.104 (4)	0.054 (2)	−0.036 (3)	−0.020 (2)	0.019 (2)
C68	0.076 (3)	0.098 (3)	0.091 (3)	−0.039 (2)	−0.042 (2)	0.042 (3)
C69	0.060 (2)	0.065 (2)	0.111 (3)	−0.0186 (18)	−0.043 (2)	0.021 (2)
C70	0.0555 (19)	0.063 (2)	0.068 (2)	−0.0200 (16)	−0.0230 (16)	0.0060 (19)
C71	0.0457 (16)	0.0379 (16)	0.0438 (16)	−0.0113 (13)	−0.0130 (13)	0.0001 (13)
C72	0.0460 (17)	0.059 (2)	0.061 (2)	−0.0135 (15)	−0.0183 (15)	−0.0031 (16)
C73	0.0456 (17)	0.063 (2)	0.062 (2)	−0.0090 (15)	−0.0032 (16)	−0.0036 (17)
C74	0.073 (2)	0.0427 (18)	0.0426 (18)	−0.0097 (16)	−0.0075 (17)	0.0001 (14)
C75	0.080 (2)	0.0490 (19)	0.054 (2)	−0.0126 (17)	−0.0315 (18)	−0.0025 (16)
C76	0.0500 (17)	0.0489 (18)	0.059 (2)	−0.0121 (14)	−0.0234 (16)	−0.0039 (15)
O13	0.115 (2)	0.112 (2)	0.0486 (15)	0.0127 (17)	−0.0069 (14)	−0.0010 (14)
C77	0.176 (6)	0.156 (5)	0.090 (3)	−0.079 (4)	−0.044 (4)	0.007 (3)
C79	0.117 (4)	0.116 (4)	0.071 (3)	−0.037 (3)	−0.033 (3)	0.024 (3)
O14	0.079 (7)	0.128 (9)	0.085 (8)	−0.016 (7)	−0.032 (6)	0.024 (7)
C78A	0.082 (7)	0.096 (8)	0.050 (4)	−0.024 (6)	−0.017 (5)	0.000 (4)
C78B	0.074 (9)	0.085 (8)	0.049 (6)	0.010 (7)	−0.003 (6)	0.003 (5)

### 2-(4-Nitrophenyl)-3-phenyl-2,3-dihydro-4H-pyrido[3,2-e][1,3]thiazin-4-one isopropanol 0.25-solvate 0.0625-hydrate (2) Geometric parameters (Å, º)

**Table:**

S1—C1	1.821 (3)	C39—C52	1.521 (4)
S1—C4	1.737 (3)	C40—C41	1.493 (4)
O1—C2	1.221 (3)	C41—C42	1.397 (4)
O2—N3	1.209 (3)	C41—C45	1.382 (4)
O3—N3	1.209 (3)	C43—H43	0.9300
N1—C1	1.466 (3)	C43—C44	1.369 (5)
N1—C2	1.369 (3)	C44—H44	0.9300
N1—C8	1.434 (3)	C44—C45	1.371 (4)
N2—C4	1.346 (3)	C45—H45	0.9300
N2—C5	1.327 (4)	C46—C47	1.376 (4)
N3—C17	1.475 (4)	C46—C51	1.378 (4)
C1—H1	0.9800	C47—H47	0.9300
C1—C14	1.511 (4)	C47—C48	1.383 (5)
C2—C3	1.492 (4)	C48—H48	0.9300
C3—C4	1.392 (3)	C48—C49	1.382 (5)
C3—C7	1.389 (4)	C49—H49	0.9300
C5—H5	0.9300	C49—C50	1.369 (5)
C5—C6	1.374 (4)	C50—H50	0.9300
C6—H6	0.9300	C50—C51	1.376 (5)
C6—C7	1.378 (4)	C51—H51	0.9300
C7—H7	0.9300	C52—C53	1.385 (4)
C8—C9	1.382 (4)	C52—C57	1.388 (4)
C8—C13	1.387 (4)	C53—H53	0.9300
C9—H9	0.9300	C53—C54	1.381 (4)
C9—C10	1.378 (4)	C54—H54	0.9300
C10—H10	0.9300	C54—C55	1.358 (4)
C10—C11	1.374 (5)	C55—C56	1.384 (4)
C11—H11	0.9300	C56—H56	0.9300
C11—C12	1.377 (5)	C56—C57	1.369 (4)
C12—H12	0.9300	C57—H57	0.9300
C12—C13	1.382 (4)	S4—C58	1.821 (3)
C13—H13	0.9300	S4—C61	1.746 (3)
C14—C15	1.390 (3)	O10—C59	1.220 (3)
C14—C19	1.385 (4)	O11—N12	1.207 (4)
C15—H15	0.9300	O12—N12	1.217 (4)
C15—C16	1.369 (4)	N10—C58	1.461 (3)
C16—H16	0.9300	N10—C59	1.367 (3)
C16—C17	1.369 (4)	N10—C65	1.443 (4)
C17—C18	1.366 (4)	N11—C61	1.332 (4)
C18—H18	0.9300	N11—C62	1.327 (4)
C18—C19	1.382 (4)	N12—C74	1.475 (4)
C19—H19	0.9300	C58—H58	0.9800
S2—C20	1.821 (3)	C58—C71	1.518 (4)
S2—C23	1.742 (3)	C59—C60	1.486 (4)
O4—C21	1.219 (3)	C60—C61	1.392 (4)
O5—N6	1.214 (3)	C60—C64	1.385 (4)
O6—N6	1.211 (3)	C62—H62	0.9300
N4—C20	1.462 (3)	C62—C63	1.365 (5)
N4—C21	1.364 (3)	C63—H63	0.9300
N4—C27	1.432 (4)	C63—C64	1.382 (4)
N5—C23	1.335 (4)	C64—H64	0.9300
N5—C24	1.332 (4)	C65—C66	1.378 (4)
N6—C36	1.484 (4)	C65—C70	1.373 (4)
C20—H20	0.9800	C66—H66	0.9300
C20—C33	1.517 (4)	C66—C67	1.377 (5)
C21—C22	1.485 (4)	C67—H67	0.9300
C22—C23	1.400 (4)	C67—C68	1.372 (5)
C22—C26	1.390 (4)	C68—H68	0.9300
C24—H24	0.9300	C68—C69	1.354 (5)
C24—C25	1.370 (4)	C69—H69	0.9300
C25—H25	0.9300	C69—C70	1.385 (4)
C25—C26	1.373 (4)	C70—H70	0.9300
C26—H26	0.9300	C71—C72	1.382 (4)
C27—C28	1.381 (4)	C71—C76	1.388 (4)
C27—C32	1.382 (4)	C72—H72	0.9300
C28—H28	0.9300	C72—C73	1.380 (4)
C28—C29	1.380 (5)	C73—H73	0.9300
C29—H29	0.9300	C73—C74	1.365 (4)
C29—C30	1.368 (5)	C74—C75	1.375 (4)
C30—H30	0.9300	C75—H75	0.9300
C30—C31	1.366 (5)	C75—C76	1.367 (4)
C31—H31	0.9300	C76—H76	0.9300
C31—C32	1.382 (4)	O13—H13A	0.8200
C32—H32	0.9300	O13—H13B	0.8200
C33—C34	1.385 (3)	O13—C78A	1.422 (8)
C33—C38	1.393 (4)	O13—C78B	1.564 (14)
C34—H34	0.9300	C77—H77A	0.9600
C34—C35	1.378 (4)	C77—H77B	0.9600
C35—H35	0.9300	C77—H77C	0.9600
C35—C36	1.372 (4)	C77—H77D	0.9600
C36—C37	1.367 (4)	C77—H77E	0.9600
C37—H37	0.9300	C77—H77F	0.9600
C37—C38	1.371 (4)	C77—C78A	1.409 (11)
C38—H38	0.9300	C77—C78B	1.512 (17)
S3—C39	1.816 (3)	C79—H79A	0.9600
S3—C42	1.744 (3)	C79—H79B	0.9600
O7—C40	1.225 (3)	C79—H79C	0.9600
O8—N9	1.209 (3)	C79—H79D	0.9600
O9—N9	1.207 (3)	C79—H79E	0.9600
N7—C39	1.464 (3)	C79—H79F	0.9600
N7—C40	1.370 (3)	C79—C78A	1.496 (10)
N7—C46	1.441 (4)	C79—C78B	1.371 (13)
N8—C42	1.338 (4)	O14—H14A	0.8499
N8—C43	1.331 (4)	O14—H14B	0.8501
N9—C55	1.468 (4)	C78A—H78A	0.9800
C39—H39	0.9800	C78B—H78B	0.9800
			
C4—S1—C1	97.70 (12)	N8—C42—S3	114.8 (2)
C2—N1—C1	122.3 (2)	N8—C42—C41	123.8 (3)
C2—N1—C8	119.9 (2)	C41—C42—S3	121.4 (2)
C8—N1—C1	117.4 (2)	N8—C43—H43	117.8
C5—N2—C4	117.3 (2)	N8—C43—C44	124.3 (3)
O2—N3—C17	118.0 (3)	C44—C43—H43	117.8
O3—N3—O2	123.6 (3)	C43—C44—H44	120.5
O3—N3—C17	118.4 (3)	C43—C44—C45	119.0 (3)
S1—C1—H1	105.8	C45—C44—H44	120.5
N1—C1—S1	112.14 (18)	C41—C45—H45	120.6
N1—C1—H1	105.8	C44—C45—C41	118.9 (3)
N1—C1—C14	115.6 (2)	C44—C45—H45	120.6
C14—C1—S1	110.95 (18)	C47—C46—N7	119.8 (3)
C14—C1—H1	105.8	C47—C46—C51	120.4 (3)
O1—C2—N1	122.1 (2)	C51—C46—N7	119.8 (3)
O1—C2—C3	120.1 (2)	C46—C47—H47	120.3
N1—C2—C3	117.8 (2)	C46—C47—C48	119.4 (4)
C4—C3—C2	124.1 (2)	C48—C47—H47	120.3
C7—C3—C2	117.8 (2)	C47—C48—H48	119.8
C7—C3—C4	117.7 (3)	C49—C48—C47	120.3 (4)
N2—C4—S1	115.1 (2)	C49—C48—H48	119.8
N2—C4—C3	123.2 (3)	C48—C49—H49	120.2
C3—C4—S1	121.7 (2)	C50—C49—C48	119.6 (4)
N2—C5—H5	118.1	C50—C49—H49	120.2
N2—C5—C6	123.9 (3)	C49—C50—H50	119.7
C6—C5—H5	118.1	C49—C50—C51	120.6 (4)
C5—C6—H6	120.7	C51—C50—H50	119.7
C5—C6—C7	118.7 (3)	C46—C51—H51	120.2
C7—C6—H6	120.7	C50—C51—C46	119.7 (3)
C3—C7—H7	120.4	C50—C51—H51	120.2
C6—C7—C3	119.2 (3)	C53—C52—C39	122.3 (2)
C6—C7—H7	120.4	C53—C52—C57	118.5 (3)
C9—C8—N1	120.9 (3)	C57—C52—C39	119.2 (2)
C9—C8—C13	119.3 (3)	C52—C53—H53	119.7
C13—C8—N1	119.8 (3)	C54—C53—C52	120.6 (3)
C8—C9—H9	119.9	C54—C53—H53	119.7
C10—C9—C8	120.2 (3)	C53—C54—H54	120.4
C10—C9—H9	119.9	C55—C54—C53	119.2 (3)
C9—C10—H10	119.8	C55—C54—H54	120.4
C11—C10—C9	120.4 (3)	C54—C55—N9	118.1 (3)
C11—C10—H10	119.8	C54—C55—C56	122.0 (3)
C10—C11—H11	120.1	C56—C55—N9	119.8 (3)
C10—C11—C12	119.8 (3)	C55—C56—H56	121.0
C12—C11—H11	120.1	C57—C56—C55	118.1 (3)
C11—C12—H12	119.9	C57—C56—H56	121.0
C11—C12—C13	120.2 (3)	C52—C57—H57	119.2
C13—C12—H12	119.9	C56—C57—C52	121.6 (3)
C8—C13—H13	120.0	C56—C57—H57	119.2
C12—C13—C8	120.1 (3)	C61—S4—C58	97.01 (14)
C12—C13—H13	120.0	C59—N10—C58	122.4 (2)
C15—C14—C1	118.0 (2)	C59—N10—C65	119.7 (2)
C19—C14—C1	123.5 (2)	C65—N10—C58	117.8 (2)
C19—C14—C15	118.5 (3)	C62—N11—C61	116.9 (3)
C14—C15—H15	119.3	O11—N12—O12	124.0 (3)
C16—C15—C14	121.5 (3)	O11—N12—C74	117.6 (4)
C16—C15—H15	119.3	O12—N12—C74	118.3 (4)
C15—C16—H16	120.8	S4—C58—H58	106.5
C17—C16—C15	118.5 (3)	N10—C58—S4	111.63 (19)
C17—C16—H16	120.8	N10—C58—H58	106.5
C16—C17—N3	118.7 (3)	N10—C58—C71	114.8 (2)
C18—C17—N3	119.3 (3)	C71—C58—S4	110.50 (18)
C18—C17—C16	122.0 (3)	C71—C58—H58	106.5
C17—C18—H18	120.4	O10—C59—N10	121.8 (3)
C17—C18—C19	119.2 (3)	O10—C59—C60	120.9 (3)
C19—C18—H18	120.4	N10—C59—C60	117.2 (3)
C14—C19—H19	119.8	C61—C60—C59	124.3 (3)
C18—C19—C14	120.4 (3)	C64—C60—C59	118.1 (3)
C18—C19—H19	119.8	C64—C60—C61	117.2 (3)
C23—S2—C20	97.49 (13)	N11—C61—S4	114.7 (2)
C21—N4—C20	121.7 (2)	N11—C61—C60	123.8 (3)
C21—N4—C27	119.5 (2)	C60—C61—S4	121.4 (2)
C27—N4—C20	118.6 (2)	N11—C62—H62	117.9
C24—N5—C23	117.0 (3)	N11—C62—C63	124.3 (3)
O5—N6—C36	118.2 (3)	C63—C62—H62	117.9
O6—N6—O5	124.1 (3)	C62—C63—H63	120.9
O6—N6—C36	117.7 (3)	C62—C63—C64	118.2 (3)
S2—C20—H20	105.9	C64—C63—H63	120.9
N4—C20—S2	112.10 (19)	C60—C64—H64	120.3
N4—C20—H20	105.9	C63—C64—C60	119.4 (3)
N4—C20—C33	115.2 (2)	C63—C64—H64	120.3
C33—C20—S2	111.0 (2)	C66—C65—N10	119.1 (3)
C33—C20—H20	105.9	C70—C65—N10	120.2 (3)
O4—C21—N4	121.1 (3)	C70—C65—C66	120.7 (3)
O4—C21—C22	120.7 (2)	C65—C66—H66	120.4
N4—C21—C22	118.2 (2)	C67—C66—C65	119.3 (4)
C23—C22—C21	123.7 (3)	C67—C66—H66	120.4
C26—C22—C21	119.2 (2)	C66—C67—H67	119.9
C26—C22—C23	116.7 (3)	C68—C67—C66	120.2 (4)
N5—C23—S2	114.6 (2)	C68—C67—H67	119.9
N5—C23—C22	123.7 (3)	C67—C68—H68	119.9
C22—C23—S2	121.6 (2)	C69—C68—C67	120.2 (4)
N5—C24—H24	118.0	C69—C68—H68	119.9
N5—C24—C25	124.0 (3)	C68—C69—H69	119.6
C25—C24—H24	118.0	C68—C69—C70	120.8 (4)
C24—C25—H25	120.8	C70—C69—H69	119.6
C24—C25—C26	118.3 (3)	C65—C70—C69	118.9 (3)
C26—C25—H25	120.8	C65—C70—H70	120.6
C22—C26—H26	120.0	C69—C70—H70	120.6
C25—C26—C22	120.0 (3)	C72—C71—C58	123.0 (2)
C25—C26—H26	120.0	C72—C71—C76	118.4 (3)
C28—C27—N4	119.6 (3)	C76—C71—C58	118.6 (2)
C28—C27—C32	119.9 (3)	C71—C72—H72	119.5
C32—C27—N4	120.5 (3)	C73—C72—C71	120.9 (3)
C27—C28—H28	120.3	C73—C72—H72	119.5
C29—C28—C27	119.4 (4)	C72—C73—H73	120.6
C29—C28—H28	120.3	C74—C73—C72	118.9 (3)
C28—C29—H29	119.7	C74—C73—H73	120.6
C30—C29—C28	120.6 (4)	C73—C74—N12	119.8 (3)
C30—C29—H29	119.7	C73—C74—C75	121.8 (3)
C29—C30—H30	119.9	C75—C74—N12	118.4 (3)
C31—C30—C29	120.2 (4)	C74—C75—H75	120.6
C31—C30—H30	119.9	C76—C75—C74	118.7 (3)
C30—C31—H31	119.9	C76—C75—H75	120.6
C30—C31—C32	120.1 (4)	C71—C76—H76	119.3
C32—C31—H31	119.9	C75—C76—C71	121.3 (3)
C27—C32—H32	120.1	C75—C76—H76	119.3
C31—C32—C27	119.8 (3)	C78A—O13—H13A	109.5
C31—C32—H32	120.1	C78B—O13—H13B	109.5
C34—C33—C20	123.3 (2)	H77A—C77—H77B	109.5
C34—C33—C38	118.5 (3)	H77A—C77—H77C	109.5
C38—C33—C20	118.2 (2)	H77B—C77—H77C	109.5
C33—C34—H34	119.6	H77D—C77—H77E	109.5
C35—C34—C33	120.9 (3)	H77D—C77—H77F	109.5
C35—C34—H34	119.6	H77E—C77—H77F	109.5
C34—C35—H35	120.7	C78A—C77—H77A	109.5
C36—C35—C34	118.6 (3)	C78A—C77—H77B	109.5
C36—C35—H35	120.7	C78A—C77—H77C	109.5
C35—C36—N6	119.6 (3)	C78B—C77—H77D	109.5
C37—C36—N6	118.2 (3)	C78B—C77—H77E	109.5
C37—C36—C35	122.2 (3)	C78B—C77—H77F	109.5
C36—C37—H37	120.6	H79A—C79—H79B	109.5
C36—C37—C38	118.8 (3)	H79A—C79—H79C	109.5
C38—C37—H37	120.6	H79B—C79—H79C	109.5
C33—C38—H38	119.5	H79D—C79—H79E	109.5
C37—C38—C33	121.0 (3)	H79D—C79—H79F	109.5
C37—C38—H38	119.5	H79E—C79—H79F	109.5
C42—S3—C39	97.62 (13)	C78A—C79—H79A	109.5
C40—N7—C39	121.1 (2)	C78A—C79—H79B	109.5
C40—N7—C46	120.7 (2)	C78A—C79—H79C	109.5
C46—N7—C39	117.3 (2)	C78B—C79—H79D	109.5
C43—N8—C42	116.2 (3)	C78B—C79—H79E	109.5
O8—N9—C55	118.8 (3)	C78B—C79—H79F	109.5
O9—N9—O8	122.5 (3)	H14A—O14—H14B	109.5
O9—N9—C55	118.7 (3)	O13—C78A—C79	110.4 (7)
S3—C39—H39	106.1	O13—C78A—H78A	103.9
N7—C39—S3	112.11 (19)	C77—C78A—O13	114.2 (8)
N7—C39—H39	106.1	C77—C78A—C79	118.5 (9)
N7—C39—C52	114.1 (2)	C77—C78A—H78A	103.9
C52—C39—S3	111.63 (19)	C79—C78A—H78A	103.9
C52—C39—H39	106.1	O13—C78B—H78B	108.6
O7—C40—N7	122.3 (3)	C77—C78B—O13	101.2 (12)
O7—C40—C41	119.4 (3)	C77—C78B—H78B	108.6
N7—C40—C41	118.3 (2)	C79—C78B—O13	109.3 (9)
C42—C41—C40	123.9 (3)	C79—C78B—C77	119.9 (10)
C45—C41—C40	118.2 (3)	C79—C78B—H78B	108.6
C45—C41—C42	117.7 (3)		
			
S1—C1—C14—C15	68.1 (3)	S3—C39—C52—C53	−108.1 (3)
S1—C1—C14—C19	−114.3 (3)	S3—C39—C52—C57	72.3 (3)
O1—C2—C3—C4	157.9 (3)	O7—C40—C41—C42	159.3 (3)
O1—C2—C3—C7	−15.1 (4)	O7—C40—C41—C45	−14.5 (4)
O2—N3—C17—C16	−163.6 (3)	O8—N9—C55—C54	9.4 (4)
O2—N3—C17—C18	17.5 (4)	O8—N9—C55—C56	−169.0 (3)
O3—N3—C17—C16	15.8 (4)	O9—N9—C55—C54	−171.6 (3)
O3—N3—C17—C18	−163.1 (3)	O9—N9—C55—C56	10.1 (4)
N1—C1—C14—C15	−162.7 (2)	N7—C39—C52—C53	20.3 (4)
N1—C1—C14—C19	14.9 (4)	N7—C39—C52—C57	−159.4 (3)
N1—C2—C3—C4	−20.3 (4)	N7—C40—C41—C42	−19.5 (4)
N1—C2—C3—C7	166.7 (2)	N7—C40—C41—C45	166.7 (2)
N1—C8—C9—C10	−179.1 (2)	N7—C46—C47—C48	179.3 (3)
N1—C8—C13—C12	178.5 (3)	N7—C46—C51—C50	179.4 (3)
N2—C5—C6—C7	3.0 (5)	N8—C43—C44—C45	3.7 (5)
N3—C17—C18—C19	178.1 (2)	N9—C55—C56—C57	178.5 (3)
C1—S1—C4—N2	−156.8 (2)	C39—S3—C42—N8	−157.9 (2)
C1—S1—C4—C3	26.2 (2)	C39—S3—C42—C41	25.0 (3)
C1—N1—C2—O1	170.8 (2)	C39—N7—C40—O7	167.4 (3)
C1—N1—C2—C3	−11.1 (4)	C39—N7—C40—C41	−13.8 (4)
C1—N1—C8—C9	134.7 (3)	C39—N7—C46—C47	−36.5 (4)
C1—N1—C8—C13	−43.3 (3)	C39—N7—C46—C51	142.6 (3)
C1—C14—C15—C16	177.7 (2)	C39—C52—C53—C54	179.7 (3)
C1—C14—C19—C18	−178.3 (2)	C39—C52—C57—C56	179.5 (3)
C2—N1—C1—S1	49.1 (3)	C40—N7—C39—S3	52.1 (3)
C2—N1—C1—C14	−79.4 (3)	C40—N7—C39—C52	−76.0 (3)
C2—N1—C8—C9	−52.5 (3)	C40—N7—C46—C47	132.7 (3)
C2—N1—C8—C13	129.5 (3)	C40—N7—C46—C51	−48.2 (4)
C2—C3—C4—S1	6.5 (4)	C40—C41—C42—S3	7.8 (4)
C2—C3—C4—N2	−170.2 (2)	C40—C41—C42—N8	−169.0 (3)
C2—C3—C7—C6	171.8 (3)	C40—C41—C45—C44	171.5 (3)
C4—S1—C1—N1	−50.9 (2)	C42—S3—C39—N7	−52.0 (2)
C4—S1—C1—C14	80.10 (19)	C42—S3—C39—C52	77.5 (2)
C4—N2—C5—C6	−1.9 (4)	C42—N8—C43—C44	−1.7 (5)
C4—C3—C7—C6	−1.7 (4)	C42—C41—C45—C44	−2.7 (4)
C5—N2—C4—S1	−178.0 (2)	C43—N8—C42—S3	−179.6 (2)
C5—N2—C4—C3	−1.1 (4)	C43—N8—C42—C41	−2.7 (4)
C5—C6—C7—C3	−1.0 (4)	C43—C44—C45—C41	−1.2 (5)
C7—C3—C4—S1	179.6 (2)	C45—C41—C42—S3	−178.4 (2)
C7—C3—C4—N2	2.9 (4)	C45—C41—C42—N8	4.9 (4)
C8—N1—C1—S1	−138.2 (2)	C46—N7—C39—S3	−138.7 (2)
C8—N1—C1—C14	93.2 (3)	C46—N7—C39—C52	93.1 (3)
C8—N1—C2—O1	−1.7 (4)	C46—N7—C40—O7	−1.4 (4)
C8—N1—C2—C3	176.5 (2)	C46—N7—C40—C41	177.4 (2)
C8—C9—C10—C11	1.0 (4)	C46—C47—C48—C49	1.4 (5)
C9—C8—C13—C12	0.5 (4)	C47—C46—C51—C50	−1.5 (4)
C9—C10—C11—C12	−0.2 (5)	C47—C48—C49—C50	−1.7 (6)
C10—C11—C12—C13	−0.5 (5)	C48—C49—C50—C51	0.5 (6)
C11—C12—C13—C8	0.4 (5)	C49—C50—C51—C46	1.1 (5)
C13—C8—C9—C10	−1.2 (4)	C51—C46—C47—C48	0.2 (5)
C14—C15—C16—C17	0.3 (4)	C52—C53—C54—C55	1.2 (4)
C15—C14—C19—C18	−0.7 (4)	C53—C52—C57—C56	−0.2 (4)
C15—C16—C17—N3	−178.8 (2)	C53—C54—C55—N9	−179.3 (2)
C15—C16—C17—C18	0.1 (4)	C53—C54—C55—C56	−1.0 (4)
C16—C17—C18—C19	−0.7 (4)	C54—C55—C56—C57	0.2 (4)
C17—C18—C19—C14	1.1 (4)	C55—C56—C57—C52	0.4 (4)
C19—C14—C15—C16	0.0 (4)	C57—C52—C53—C54	−0.7 (4)
S2—C20—C33—C34	123.0 (3)	S4—C58—C71—C72	124.5 (2)
S2—C20—C33—C38	−57.6 (3)	S4—C58—C71—C76	−57.2 (3)
O4—C21—C22—C23	−157.5 (3)	O10—C59—C60—C61	−156.1 (3)
O4—C21—C22—C26	15.0 (4)	O10—C59—C60—C64	16.3 (4)
O5—N6—C36—C35	−173.3 (3)	O11—N12—C74—C73	6.5 (5)
O5—N6—C36—C37	8.4 (4)	O11—N12—C74—C75	−171.6 (3)
O6—N6—C36—C35	6.9 (4)	O12—N12—C74—C73	−173.4 (3)
O6—N6—C36—C37	−171.5 (3)	O12—N12—C74—C75	8.6 (4)
N4—C20—C33—C34	−5.7 (4)	N10—C58—C71—C72	−2.8 (4)
N4—C20—C33—C38	173.7 (2)	N10—C58—C71—C76	175.5 (2)
N4—C21—C22—C23	20.9 (4)	N10—C59—C60—C61	21.7 (4)
N4—C21—C22—C26	−166.6 (2)	N10—C59—C60—C64	−165.9 (2)
N4—C27—C28—C29	179.8 (3)	N10—C65—C66—C67	178.4 (3)
N4—C27—C32—C31	−179.2 (3)	N10—C65—C70—C69	−178.0 (3)
N5—C24—C25—C26	−4.6 (5)	N11—C62—C63—C64	−4.4 (5)
N6—C36—C37—C38	176.7 (2)	N12—C74—C75—C76	177.7 (3)
C20—S2—C23—N5	158.5 (2)	C58—S4—C61—N11	156.2 (2)
C20—S2—C23—C22	−24.2 (2)	C58—S4—C61—C60	−26.0 (3)
C20—N4—C21—O4	−168.9 (2)	C58—N10—C59—O10	−170.6 (3)
C20—N4—C21—C22	12.7 (4)	C58—N10—C59—C60	11.6 (4)
C20—N4—C27—C28	−122.6 (3)	C58—N10—C65—C66	−119.8 (3)
C20—N4—C27—C32	56.4 (3)	C58—N10—C65—C70	58.7 (4)
C20—C33—C34—C35	177.5 (3)	C58—C71—C72—C73	178.1 (3)
C20—C33—C38—C37	−177.9 (3)	C58—C71—C76—C75	−178.6 (3)
C21—N4—C20—S2	−51.3 (3)	C59—N10—C58—S4	−51.2 (3)
C21—N4—C20—C33	76.9 (3)	C59—N10—C58—C71	75.6 (3)
C21—N4—C27—C28	62.0 (4)	C59—N10—C65—C66	63.2 (4)
C21—N4—C27—C32	−119.1 (3)	C59—N10—C65—C70	−118.4 (3)
C21—C22—C23—S2	−9.0 (4)	C59—C60—C61—S4	−8.1 (4)
C21—C22—C23—N5	168.0 (3)	C59—C60—C61—N11	169.4 (3)
C21—C22—C26—C25	−171.3 (2)	C59—C60—C64—C63	−172.3 (3)
C23—S2—C20—N4	51.1 (2)	C61—S4—C58—N10	52.1 (2)
C23—S2—C20—C33	−79.2 (2)	C61—S4—C58—C71	−76.9 (2)
C23—N5—C24—C25	1.9 (4)	C61—N11—C62—C63	2.2 (5)
C23—C22—C26—C25	1.7 (4)	C61—C60—C64—C63	0.7 (4)
C24—N5—C23—S2	−179.9 (2)	C62—N11—C61—S4	179.4 (2)
C24—N5—C23—C22	2.9 (4)	C62—N11—C61—C60	1.7 (4)
C24—C25—C26—C22	2.6 (4)	C62—C63—C64—C60	2.8 (5)
C26—C22—C23—S2	178.3 (2)	C64—C60—C61—S4	179.4 (2)
C26—C22—C23—N5	−4.6 (4)	C64—C60—C61—N11	−3.1 (4)
C27—N4—C20—S2	133.4 (2)	C65—N10—C58—S4	131.9 (2)
C27—N4—C20—C33	−98.5 (3)	C65—N10—C58—C71	−101.4 (3)
C27—N4—C21—O4	6.4 (4)	C65—N10—C59—O10	6.3 (4)
C27—N4—C21—C22	−172.0 (2)	C65—N10—C59—C60	−171.5 (2)
C27—C28—C29—C30	−0.9 (5)	C65—C66—C67—C68	−0.8 (6)
C28—C27—C32—C31	−0.3 (4)	C66—C65—C70—C69	0.4 (5)
C28—C29—C30—C31	0.4 (6)	C66—C67—C68—C69	1.3 (6)
C29—C30—C31—C32	0.1 (5)	C67—C68—C69—C70	−0.9 (5)
C30—C31—C32—C27	−0.2 (5)	C68—C69—C70—C65	0.1 (5)
C32—C27—C28—C29	0.9 (5)	C70—C65—C66—C67	0.0 (5)
C33—C34—C35—C36	0.5 (4)	C71—C72—C73—C74	0.3 (4)
C34—C33—C38—C37	1.5 (4)	C72—C71—C76—C75	−0.3 (4)
C34—C35—C36—N6	−177.0 (2)	C72—C73—C74—N12	−178.1 (3)
C34—C35—C36—C37	1.3 (4)	C72—C73—C74—C75	−0.1 (5)
C35—C36—C37—C38	−1.7 (4)	C73—C74—C75—C76	−0.3 (5)
C36—C37—C38—C33	0.2 (4)	C74—C75—C76—C71	0.5 (4)
C38—C33—C34—C35	−1.9 (4)	C76—C71—C72—C73	−0.1 (4)

### 2-(4-Nitrophenyl)-3-phenyl-2,3-dihydro-4H-pyrido[3,2-e][1,3]thiazin-4-one isopropanol 0.25-solvate 0.0625-hydrate (2) Hydrogen-bond geometry (Å, º)

**Table:**

*D*—H···*A*	*D*—H	H···*A*	*D*···*A*	*D*—H···*A*
C1—H1···O13	0.98	2.49	3.461 (4)	172
C6—H6···N5^i^	0.93	2.74	3.431 (4)	132
C39—H39···O14^ii^	0.98	2.41	3.339 (10)	158
C44—H44···N11^iii^	0.93	2.77	3.479 (4)	134
C48—H48···O14	0.93	2.29	3.065 (12)	141
C50—H50···O2^iv^	0.93	2.62	3.412 (5)	144
C58—H58···O7^v^	0.98	2.49	3.246 (3)	134
C76—H76···O7^v^	0.93	2.78	3.498 (4)	135
C79—H79*A*···O9^ii^	0.96	2.57	3.353 (6)	138
C79—H79*E*···O8^vi^	0.96	2.61	3.455 (5)	147
C79—H79*F*···O2^iv^	0.96	2.75	3.493 (6)	135
O13—H13*B*···O4^vii^	0.82	1.95	2.766 (3)	176

Symmetry codes: (i) *x*, *y*, *z*−1; (ii) −*x*+1, −*y*+1, −*z*+1; (iii) *x*, *y*, *z*+1; (iv) −*x*, −*y*, −*z*+1; (v) −*x*, −*y*+1, −*z*+1; (vi) *x*, *y*−1, *z*; (vii) −*x*+1, −*y*, −*z*+1.
